# Sustained release of dermal papilla-derived extracellular vesicles from injectable microgel promotes hair growth

**DOI:** 10.7150/thno.39566

**Published:** 2020-01-01

**Authors:** Yuxin Chen, Junfei Huang, Ruosi Chen, Lunan Yang, Jin Wang, Bingcheng Liu, Lijuan Du, Yanhua Yi, James Jia, Yanwei Xu, Qian Chen, Djakaya Guydidier Ngondi, Yong Miao, Zhiqi Hu

**Affiliations:** 1 Department of Plastic and Aesthetic Surgery Nanfang Hospital of Southern Medical University Guangzhou, Guangdong Province, 510515, China.; 2 Central laboratory of the Southern Medical University, Guangzhou, Guangdong Province, 510515, China.

**Keywords:** extracellular vesicles, oxidized sodium alginates, dermal papilla, hair regrowth, sustained release.

## Abstract

Hair regeneration has long captured researchers' attention because alopecia is a common condition and current therapeutic approaches have significant limitations. Dermal papilla (DP) cells serve as a signaling center in hair follicles and regulate hair formation and cycling by paracrine secretion. Secreted EVs are important signaling mediators for intercellular communication, and DP-derived extracellular vesicles (DP-EVs) may play an important role in hair regeneration. However, the instability of EVs *in vivo* and their low long-term retention after transplantation hinder their use in clinical applications.

**Methods**: Human DP-EVs were encapsulated in partially oxidized sodium alginate (OSA) hydrogels, yielding OSA-encapsulated EVs (OSA-EVs), which act as a sustained-release system to increase the potential therapeutic effect of DP-EVs. The ability of the OSA-EVs to protect protein was assessed. The hair regeneration capacity of OSA-EVs, as well as the underlying mechanism, was explored in hair organ culture and a mouse model of depilation.

**Results**: The OSA-EVs were approximately 100 μm in diameter, and as the hydrogel degraded, DP-EVs were gradually released. In addition, the hydrogel markedly increased the stability of vesicular proteins and increased the retention of EVs *in vitro* and *in vivo*. The OSA-EVs significantly facilitated proliferation of hair matrix cells, prolonged anagen phase in cultured human hairs, and accelerated the regrowth of back hair in mice after depilation. These effects may be due to upregulation of hair growth-promoting signaling molecules such as Wnt3a and β-catenin, and downregulation of inhibitory molecule BMP2.

**Conclusion**: This study demonstrated that OSA hydrogels promote the therapeutic effects of DP-EVs, and indicate that our novel OSA-EVs could be used to treat alopecia.

## Introduction

Hair loss, also known as alopecia, is characterized by shorter anagen and longer telogen phases of hair follicles 1,2. This may be due to loss of follicular stem cell activity and an imbalance between positive and negative signaling mediators in the skin. Currently, the majority of treatments for hair loss consist of medication or hair transplantation; however, both of these approaches have limitations 2,3. For example, drugs such as finasteride and minoxidil appear ineffective in some patients and have undesirable side effects, and hair transplantation is always restricted by the availability of donor hair follicles. Therefore, it is important to explore new approaches for alopecia therapy.

Hair follicles are epidermal appendages that contain both epithelial and mesenchymal compartments, and interactions between the epithelial and mesenchymal cells regulate the cycle of hair growth 4,5. The dermal papilla (DP), a dedicated stem cell niche derived from the mesenchyme, is widely recognized as the center that triggers hair cycling by a paracrine signaling mechanism 6,7. For example, in early anagen phase, DP cells (DPCs) secrete activators such as members of the wingless-type MMTV integration site family (WNT) and transforming growth factor-beta (TGF-β) signaling pathway to upper epithelial compartments 8-10. After receiving appropriate signals, follicular epithelial cells in the hair bulb, known as matrix cells, undergo rapid proliferation and then differentiate to form the hair fiber and follicular root sheath cells 9,10. DPCs are also an important target in the pathogenesis of hair loss. In androgen-sensitive DPCs of a balding scalp, the androgen ligand-androgen receptor (DHT-AR) complex inhibits the Wnt/β-catenin signaling pathway, which alters secretion of signaling molecules and prevents hair follicles in telogen from entering into anagen phase 9.

Previous research suggests that DPCs may have potential for use in hair loss therapy. Many studies have shown that implanted DPCs can induce the growth of new hair follicles in rodents when mixed with infantile keratinocytes in a specific proportion 11-14. We previously reported that microenvironmental reprogramming by three-dimensional (3D) culture 15 or LBL 16 encapsulation enables DPCs to induce regeneration of human and mice hair follicles. Furthermore, experiments in rodents showed that cells derived from the DP can incorporate into existing DPs, and give rise to stronger hair follicles 17,18. However, several challenges must be overcome before DPCs can be used in clinical applications. First, their ability to induce hair growth decreases during *in vitro* expansion 11,19. Second, DPC acquisition is restricted by the availability of donor hair and ethical concerns 2. Finally, cell transplantation therapy in general is associated with the risk of tumor formation and immunological rejection 20,21.

Extracellular vesicles (EVs) are nanovesicles that can originate from various cell types and carry different kinds of regulatory proteins, mRNAs, and microRNAs (miRNAs). They play a significant role in regulating cell-to-cell communication, ultimately affecting physiological and pathological conditions 22,23. Recently, several studies suggested that EVs derived from stem cells exhibited notably beneficial functions similar to their mother cells. In the field of dermatology, EVs from mesenchymal stem cells (MSCs) 24-26, urine-derived stem cells (USCs) 27 and keratinocyte 28 were confirmed to modulate melanocyte pigmentation, promote wound healing, and inhibit scar formation. Mesenchymal stem cells (MSCs) 29,30 and DP-derived EVs (DP-EVs) 31-33 were shown to facilitate hair growth by activating Wnt/β-catenin signaling. Dermal fibroblasts-derived EVs do not promote hair growth in cultured human hair follicles unless stimulated by bFGF and PDGF-AA 34. In addition, the use of EVs for therapeutic treatment offers specific advantages compared with stem cell therapy.

However, the instability and low long-term retention of EVs have hindered the development of EV-based treatments. *In vivo*, EVs were cleared or internalized by surrounding cells within 72 hours after subcutaneous injection 29. Moreover, the bioactivity of molecules in EVs, including growth factors and miRNAs, may decrease over time in physiological conditions 35,36. These issues led us to expand the application of EVs through investigating biological materials for cloaking EVs, which could theoretically improve their stability and retention, ultimately improve the long-term therapeutic effect of sustained released DP-EVs.

Alginate, which is composed of the uronic acids D-mannuronic acid (M) and L-guluronic acid (G), is one of the most important biological scaffold materials. Because of its good biocompatibility and low cytotoxicity, alginate hydrogel is widely used in the biological engineering and pharmaceutical industries for cell encapsulation and drug release 37. However, alginate is not effectively degraded in mammals, and complete removal of alginate hydrogels from implantation sites takes months.^30^, ^31^ Moreover, alginate exhibits poor cell adhesion and infiltration 38.

Recently, oxidized sodium alginate (OSA) has aroused considerable attention 39. OSA hydrogel degrades more rapidly and contains more reactive groups than native alginate hydrogel 38,39. Many studies have described the positive effects of OSA and its applications for engineering tissues including bone, cartilage, blood vessel, cornea, and other soft tissues 40-43. We hypothesized that OSA hydrogel with a low degree of oxidation (DO) could provide a stable environment for EVs and enhance their beneficial effects on hair growth.

**Scheme 1:**
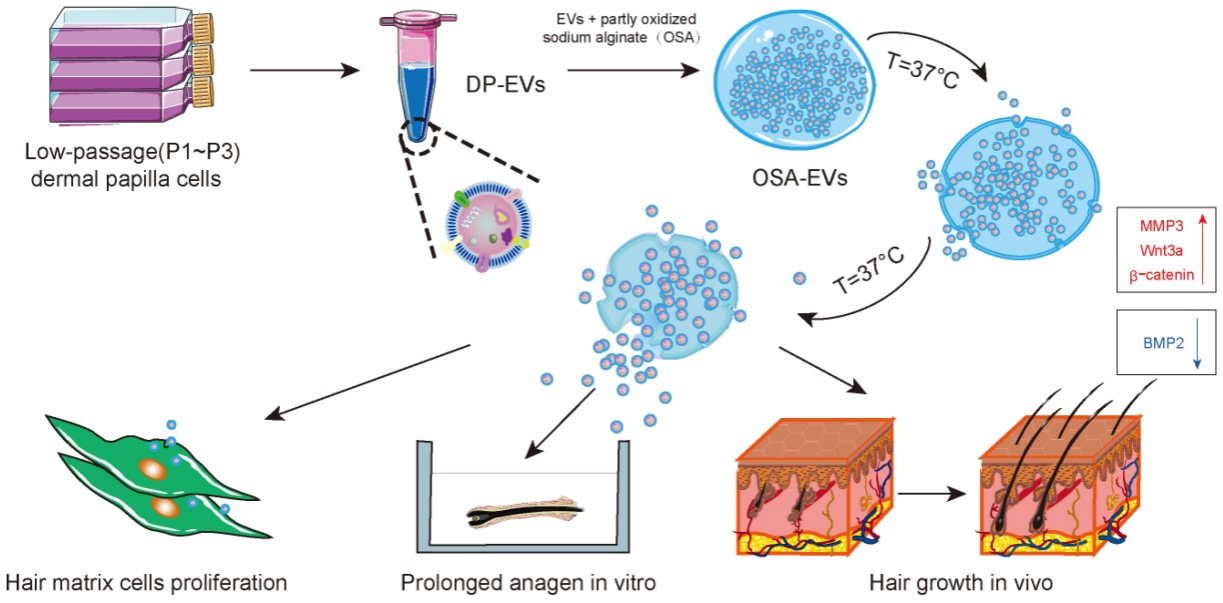
** Schematic of the preparation and functional mechanism of OSA-EV nanospheres.** EVs were isolated from low-passage human dermal papilla cells (P1-P3) and encapsulated with OSA hydrogels using a microinjection system. OSA-EV microgels were biodegradable and provided sustained release of DP-EVs. DP-EVs were absorbed by hair matrix cells, resulting in cell proliferation, prolonged anagen *in vitro*, and hair growth in vivo. The therapeutic effect of the EVs is attributed to upregulation of MMP3, Wnt3a, and β-catenin and downregulation of BMP2.

In the current study, we isolated EVs from human (h)DPs and demonstrated their therapeutic effect *in vitro* and *in vivo*. We then incorporated EVs with partially OSA hydrogels to form injectable microgels, and examined the retention and stability of EVs and OSA hydrogel-encapsulated EVs (OSA-EVs) by confocal microscopy and quantitation of protein levels. The beneficial effects of EVs and OSA-EVs were evaluated in hair matrix cells, cultured hair follicles, and a mouse model of hair loss in which the back hair is depilated. The retention of labeled EVs was examined using *in vivo* fluorescence imaging. We also investigated the signaling changes underlying the observed therapeutic effects of our EV treatment using immunofluorescence and reverse transcription polymerase chain reaction (RT-PCR) **(Scheme 1)**.

## Results and Discussion

### Isolation and Characterization of DP-EVs

**Figure 1:**
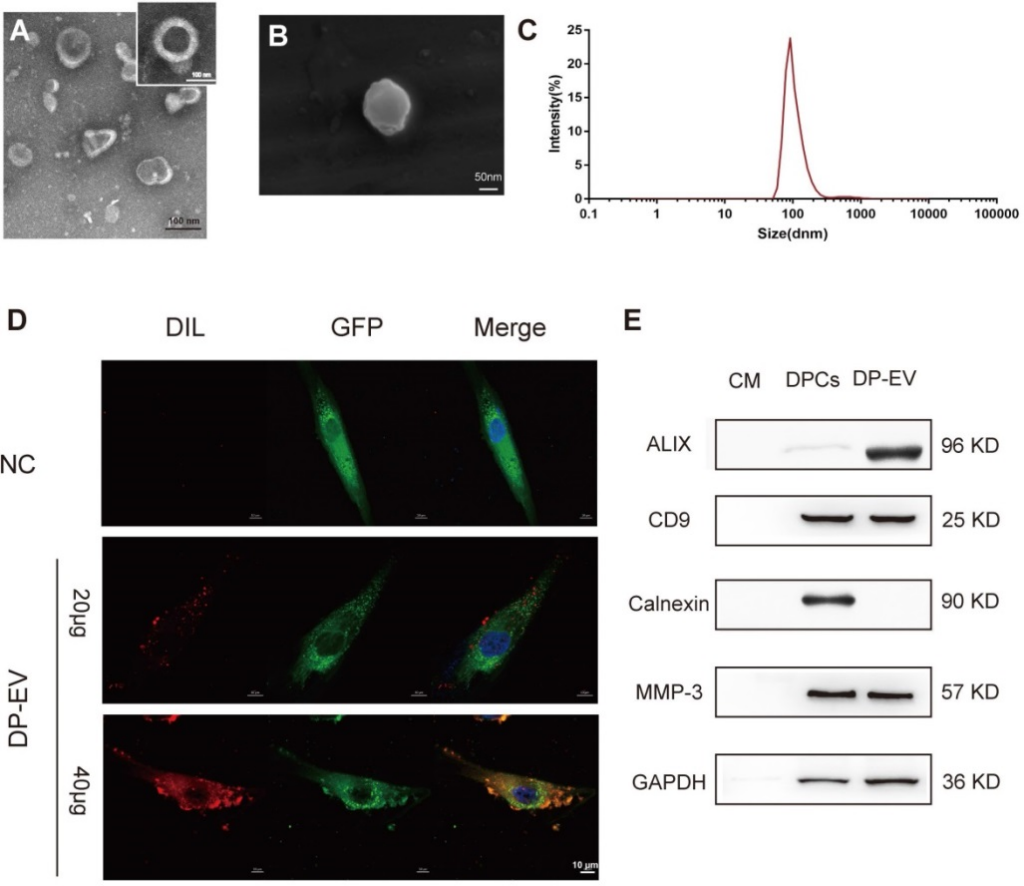
** Characterization of human dermal papilla-derived EVs.** (A) Transmission electron microscopy images of DP-EVs (scale bar: 100 nm). (B) Scanning electron microscopy images of DP-EVs (scale bar: 50 nm). (C) vesicular size distribution measured by NTA. (D) DP-EVs were labeled with DIL and were taken up by GFP-transfected hair matrix cells, with PBS as a negative control (scale bar: 10 μm). (E) Western blot of ALIX, CD9, calnexin, and MMP3 in DP cell medium, DPCs, and DP-EVs. NC: negative control.

EVs were isolated from the growth medium of low-passage (P1-P3) human hair DPC cultures as previously described 29,44. Briefly, EVs were isolated by serial centrifugation and dissolved in PBS for identification **(Figure 1)**. The DP-EVs obtained in this manner were spherically shaped and appeared to have closed membranes **(Figure 1A-B)**. The DP-EVs were investigated by nanoparticle tracking analysis (NTA), which revealed that the diameter of DP-EVs ranged from 58.8 to 255.0 nm, with an average diameter of 91.3 ± 18 nm **(Figure 1C)**. EV-specific markers were detected by western blotting **(Figure 1E)**. The surface markers CD9 and ALIX were detected, whereas the cell compartment marker calnexin was not. Based on previous studies, the signaling protein matrix metallopeptidase 3 (MMP3) is highly expressed in low-passage DPCs 45 and cooperates with Wnt3a to increase the transcriptional activity of β-catenin in C57MG cells 46; consistent with this, we found that MMP3 was expressed in DPCs and present in DP-EVs** (Figure 1E)**. To verify that DP-EVs could be taken up by hair matrix cells, GFP-transfected hair matrix cells at passage 3 (P3) were cultured with DIL-labeled EVs for 24 hours. Confocal laser scanning microscopy revealed that absorption of DP-EVs by hair matrix cells increased in a dose-dependent manner **(Figure 1D)**. These results confirmed that EVs were successfully isolated from hDPCs and could be assimilated by hair matrix cells. In order to testify the specific effect caused by DP-EVs treatment in the following experiments, we also isolated EVs from dermal fibroblasts (DF) and epidermal keratinocytes (KC). These cells are involved in the epidermis and dermis of the skin respectively. DF-EVs and KC-EVs were characterized by TEM, Western-blot and celluar uptake assay (**Figure S1**).

### DP-EVs promote proliferation and migration of hair matrix cells

**Figure 2:**
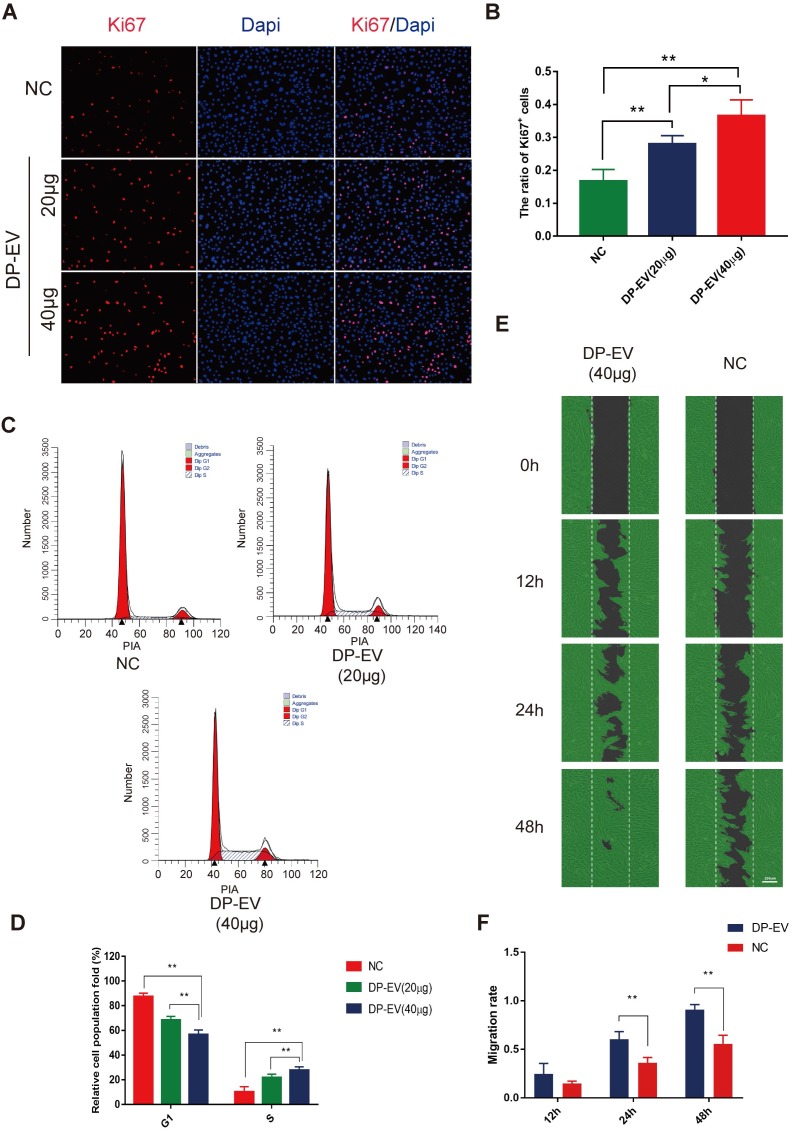
** DP-EVs promote proliferation and migration of hair matrix cells. (A)** Immunofluorescence microscopy of Ki67 in hair matrix cells treated with 20 μg DP-EVs, 40 μg DP-EVs, or PBS for 48 h. **(B)** Quantitative analysis of the percentage of Ki67^+^ cells in Figure 2A. **(C,D)** Flow cytometry profile of the cell cycle of hair matrix cells after incubation with 20 μg DP-EVs, 40 μg DP-EVs, or PBS for 48 h. (D) Quantification of hair matrix cells in S and G1 phases in the presence or absence of DPC-EVs. **(E)** Representative images of hair matrix cells in the wounded area during the migration assay, after incubation for 0, 12, 24 h and 48h with 40 ug DP-EVs or PBS. **(F)** Quantitative analysis of the decrease in wound area in Figure 2E. ** indicates a statistically significant difference (p < 0.01); all values are expressed as means ± S.D. (n = 3 individual experiments).

Hair matrix cells, a type of transit-amplifying cells (TACs), proliferate and differentiate to form the hair fiber and follicular root sheath 47. We added DP-EVs to cultures of hair matrix cells and observed their effects on growth and differentiation. The number of EVs added was calculated based on quantification of total vesicular protein (20 or 40 µg/well). To testify the specific effect caused by DP-EVs treatment, and PBS was used as control. DP-EVs promoted the proliferation of hair matrix cells in a dose-dependent manner, as determined by CCK-8 assay **(Figure S2A)** and microscopy **(Figure S2B)**. Immunofluorescence staining for Ki67, a cellular marker of proliferation, revealed that hair matrix cells treated with 40 µg DP-EVs had the highest proportion of Ki67-positive cells **(Figure 2A-B)**. Treatment with 40 μg DP-EVs caused a 2.7-fold increase in the size of the S-phase fraction relative to the PBS control and a 1.3-fold increase relative to treatment with 20 μg DP-EVs **(Figure 2C-D).** But no signicificant improvement caused by DF-EV and KC-EV treatment (**Figure S3A-B**). *In vitro* migration assays revealed that DP-EV-treatment promoted wound healing: after 48 hours of DP-EV treatment, wound area was in average ~5.0-fold smaller than in the negative control **(Figure 2E-F)**.

To further testify whether the therapeutic effect of DP-EV-treatment was specific, we tested if DF-EVs or KC-EVs have similar effects on cell proliferation and migration (**Figure S3**). The results of cell cycle assay indicated that DP-EV-treatment caused a ~1.9-fold increase in the size of the S-phase fraction relative to the PBS control, but no signicificant improvement was caused by DF-EV- and KC-EV-treatment. For migration assays, wound area was in average ~5.0-fold smaller than that of the negative control after 48 hours of DP-EVs treatment. No significant difference was found between DF-EV-, KC-EV-treated group and NC group (**Figure S3C-D**). These results demonstrated that DP-EVs specifically stimulate the proliferation and migration of hair matrix cells.

### Preparation and characterization of OSA hydrogel

**Figure 3:**
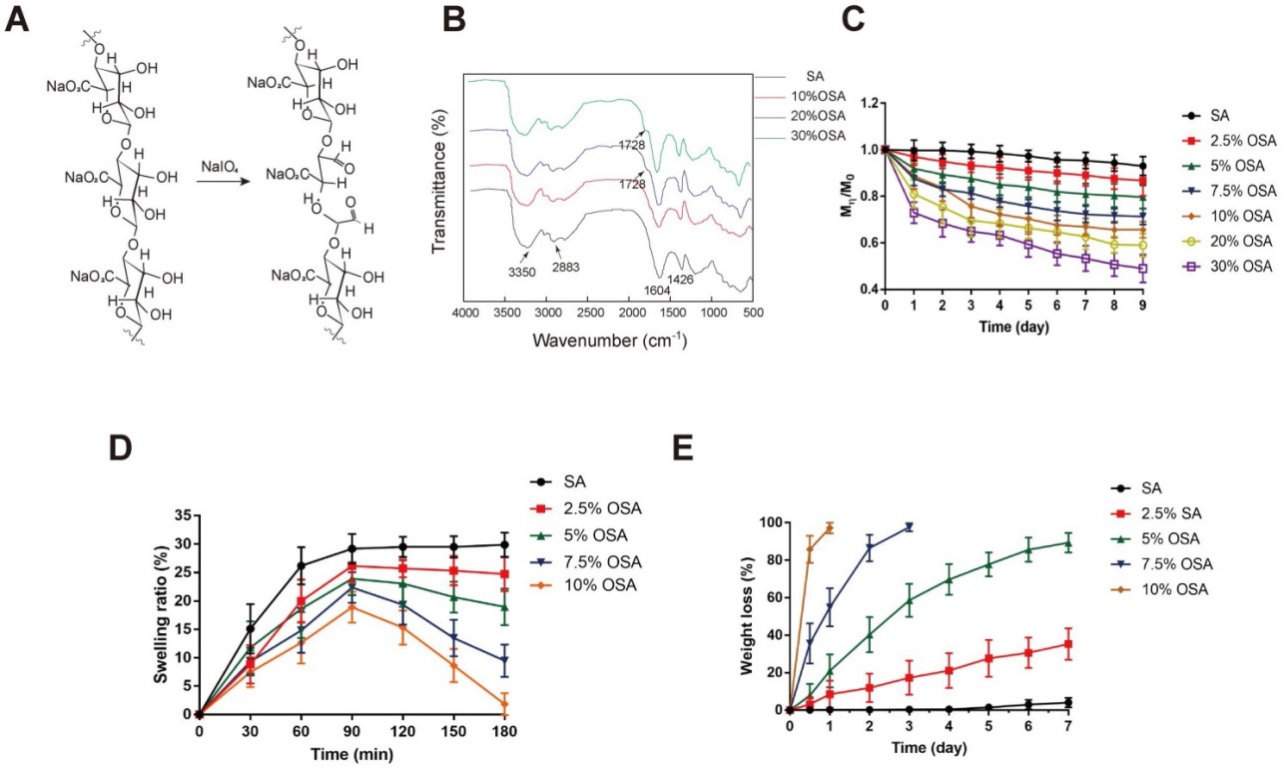
** Preparation and characterization of OSA hydrogel.** (A) A possible mechanism for the oxidation reaction of sodium alginate by periodate. (B) FTIR spectra of OSA with 10%, 20%, and 30% DO. (C) Degradation kinetics of native SA and 2.5%, 5%, 7.5%, 10%, 20%, and 30% oxidized OSA in PBS (pH 7.4), at 37°C. (D) Swelling kinetics of native SA and 2.5%, 5%, 7.5%, and 10% OSA hydrogels in PBS (pH 7.4) at 37°C.(E) Degradation kinetics of OSA hydrogels in PBS (pH 7.4), at 37°C.

OSA hydrogel is a degradable biomaterial that has been used in a wide variety of tissue engineering applications 40-43. Many reports suggest that the swelling and degradation of OSA hydrogel depends on the DO 38,39,41. However, the use of OSA still elicits debate in light of the potential cytotoxicity caused by aldehyde groups that are present in the polymer chains following alginate oxidation. Gao et al. demonstrated that OSA with relatively low DO (≤30%) has very low toxicity 38,39,41. To determine which DO of sodium alginate was best suited for coating EVs, we prepared OSA with DO of 2.5%, 5%, 7.5%, 10%, 20% and 30%, using sodium periodate as previously described with slight modifications 39,48 **(Table 1)**. Consistent with previous studies, higher DO was associated with lower molecular mass. In mechanistic terms, alginate oxidation by periodate may occur via diol (C-C) cleavage and hemiacetal formation 38 **(Figure 3A)**, and the DO and molecular weight depend on the ratio of periodate to alginate **(Table 1)**. As in previous studies 38,48,49, we evaluated formation of aldehyde groups in alginate chains induced by periodate by Fourier transform infrared (FTIR) spectroscopy **(Figure 3B)**. Compared with the spectrum of native alginate, the spectra of oxidized alginates exhibited characteristic new aldehyde peaks in the 1740-1720 cm^-1^ region.

However, only OSA with 20% and 30% DO exhibited a new peak corresponding to the aldehyde vibration at 1728 cm^-1^, which was not detected in spectra of OSA with DO < 10% DO. The increase in aldehyde groups in OSA depends on an increase in DO 40,48. Accordingly, OSA with a low DO may yield in an inconspicuous band that is obscured by the original wave. The dynamic degradation of OSA is shown in **Figure 3C**. Degradation was assessed by calculating the percentage of the initial mass that remained after 9 days. OSA with a higher DO exhibited a faster degradation rate and a premature plateau, whereas the molecular weight of native alginate was nearly unchanged over 9 days of observation. On day 9, the percentages remaining of native alginate and OSA with DO of 2.5%, 5%, 7.5%, 10%, 20%, and 30% OSA were 93.0%, 86.7%, 79.7%, 71.3%, 65.7%, 59.0%, and 49.0%, respectively.

Crosslinking of alginate with Ca^2+^ initiates hydrogel formation. However, we found that OSA with DO of 20% or 30% DO did not form hydrogels when we attempted the crosslinking reaction. This is due to the relatively low molecular weight of 20% and 30% OSA, and the associated reduction in the cooperative interaction between carboxylate groups and Ca^2+^ 50. In the first 30 minutes of incubation, the SR of all groups increased, indicating that water molecules were diffusing into the gel network and some Na^+^ ions were taking the place of Ca^2+^; however, the degree of replacement was not enough to break the scaffold. The higher the crosslinking density of a hydrogel, the lower the SR 51. The peaks of the SR curve for native alginate and 2.5%, 5%, 7.5%, and 10% OSA were 29.9%, 26.1%, 23.9%, 22.4%, and 18.9%, respectively **(Figure 3D)**.

With prolonged incubation, more calcium ions were replaced, and when equilibrium was reached, the scaffold started to degrade, as reflected by a decrease in the swelling curve. In addition, as a consequence of the increasing flexibility of C-C bonds, the chain was easier to hydrolyze 38. High-DO OSAs exhibited a more rapidly decreasing swelling curve, which can be attributed to their lower crosslinking density and higher chain flexibility **(Figure 3D)**. The weight of 10% and 7.5% OSAs decreased to an undetectable level on days 1 and 3, respectively **(Figure 3E)**. The weight loss percentages of native alginate and 2.5% and 5% OSA hydrogels on day 7 were 4.07%, 35.27%, and 89.38%, respectively, indicating that higher DO leads to faster hydrogel degradation.

**Figure 4:**
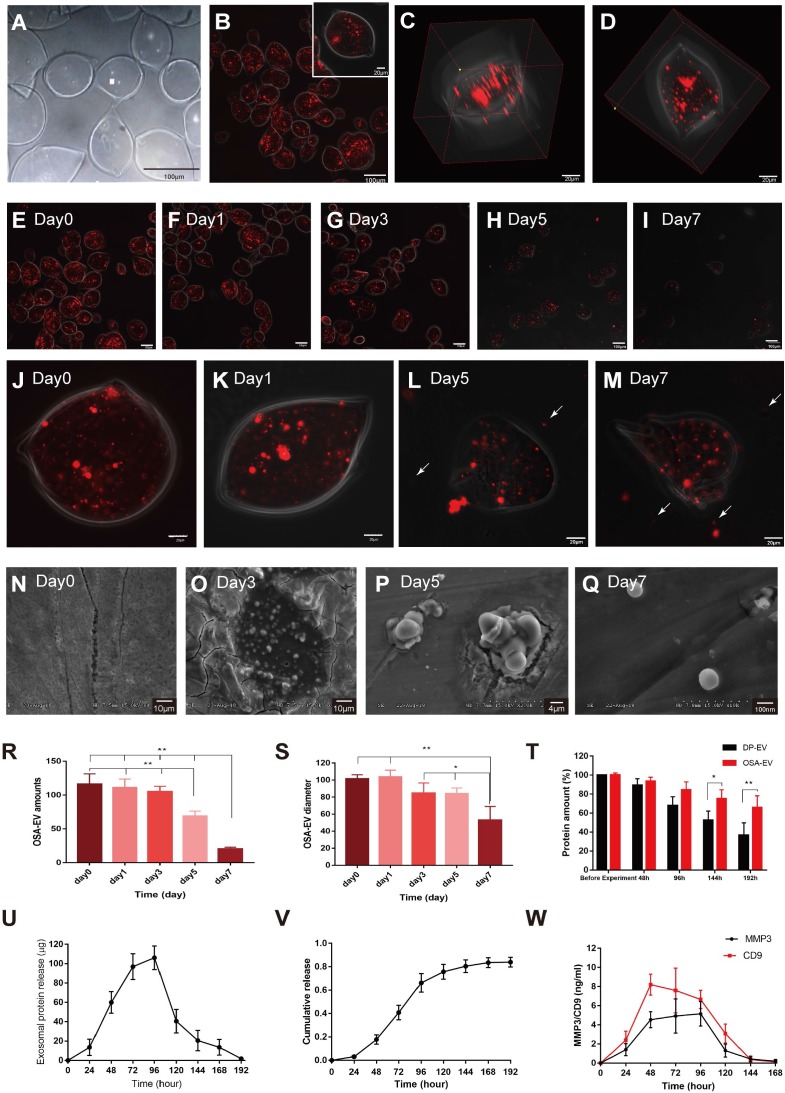
** OSA-EV hydrogel protects DP-EVs from degradation and permits their sustained release.** (A-C) DP-EVs encapsulated with 5% DO OSA hydrogel. Bright field (A) and confocal microscopy images (B) of OSA-EV microgels with OSA hydrogel nanofilm-coated, DIL-labeled DP-EVs. Scale bars: 100 μm. (C) 3D reconstruction of OSA-EV nanosphere is projected from various angles. Scale bars: 20 μm. (E-M) Confocal microscopic images of OSA-EV microgels over a period of 7 days. OSA-EV microgels change with time at low (E-I) and high magnification (J-M); E-I, scale bars: 100 μm; J-M, scale bars: 20 μm. (N-Q) SEM images of OSA-EV nanofilms captured at days 0, 3, 5, and 7. (R) The amount of OSA-EV microgels was calculated from confocal microscopy images. (S) Changes in the diameter of OSA-EV microgels were calculated from confocal microscopy images. (T) Quantification of total protein after incubating OSA-EVs in PBS pH 7.4 for 192 hours at 37°C confirmed that the nanofilm protected vesicular proteins even as the nanofilm degraded. (U) *In vitro* dynamic release of vesicular proteins from OSA-EVs, quantified by BCA protein assay. (V) Cumulative release data of vesicular total proteins from OSA-EV hydrogel microgels. (W) Release of vesicular proteins MMP3 and CD9 from OSA-EV microgels after incubating in PBS pH 7.4 for 168 hours at 37°C, as determined by ELISA. * indicates statistically significant difference, *p* < 0.05; ** indicates statistically significant difference, *p* < 0.01; all values are expressed as the mean ± S.D. (n = 3 individual experiments).

EVs are temperature-sensitive and must be stored in a low-temperature environment in order to maintain their biological activity. To ensure minimal loss of activity, the International Society for Extracellular Vesicles (ISEV) recommends storage of EVs in PBS at -80°C 36,52. EVs are easily deactivated: the miRNA and protein contents of EVs are gradually lost during storage at 4°C, as well as at -80°C 36,52. Storage at 20°C for 7 days significantly decreases EV number and antibacterial efficacy 54. When stored at 37°C for longer than 8 days, the level of vesicular protein decreases dramatically 50. Our data confirmed that the level of vesicular protein decreased to 21.08% of the initial amount during a 96-hour incubation at 37°C **(Figure 4T)**. To achieve the release of biologically active EVs, we chose 5% OSA for encapsulation of DP-EVs, which disintegrated almost completely after 7 days **(Figure 3)**.

### Encapsulation of DP-EVs with OSA hydrogel

DP-EVs were incorporated into 5% OSA hydrogel microgels of injectable size using an Eppendorf TransferMan NK2 micromanipulator **(Figure S2)**. In brief, the desired quantity of DP-EVs was mixed with OSA and dropped into CaCl_2_ solution with a microinjection tip with a 40-μm inner diameter **(Figure S2)**. After formation of the microgels, the CaCl_2_ solution was replaced with DMEM and observed. The average diameter of DIL-labeled OSA-EVs was 103 ± 74 μm **(Figure 4A-D).** Three-dimensional (3D) object reconstruction revealed that the shape of the OSA-EVs was a flat disc **(Figure 4C-D)**.

### Sustained release of DP-EVs from OSA hydrogels

OSA-EV microgels were incubated in DMEM at 37°C under a controlled atmosphere of 5% CO_2_ and 95% relative humidity, and observed for 7 days. At the beginning of the incubation, we estimated the total number of microgels by counting in three microscopic fields; the average number was 116.3 ± 15.04. After 7 days of incubation, the total number of microgels decreased to 20.33 ± 2.52 **(Figure 4E-I, R)**. Over 5 days of incubation, the mean diameter of OSA-EV microgels declined gently, then decreased dramatically at day 7 **(Figure 4J-M, S)**. The nanofilm of OSA-EVs was continuous and smooth at the beginning of incubation **(Figure 4J-M, T)**, but became more and more discontinuous and fractured over the following 3 days, and many dissociated DIL-positive dots **(Figure 4H-I, L-M, arrow)** were observed. These DIL-positive dots were approximately 100 nm in diameter, and were considered to represent DIL-labeled DP-EVs. SEM imaging revealed that on day 0, the integrity of the nanofilm of OSA-EV hydrogel was intact, with no evidence of cracks **(Figure 4N)**, but subsequently the nanofilm shrunk dramatically shrank and appeared to collapse at day 3 **(Figure 4O)**. Residues containing DP-EVs and hydrogels were obtained at day 5 **(Figure 4P)**. Upon degradation of OSA, dissociated DP-EVs were also obtained at day 7 **(Figure 4Q)**.

In addition, we tested the drug loading and encapsulation efficiency of OSA-EVs using the method described above. Drug loading efficiency was approximately 4.3%, and the encapsulation efficiency was approximately 78.5%, when the concentrations of EVs and OSA were restricted to 2.5 mg/ml and 1.5 g/ml, respectively **(Table 2)**.

We tested the stability of DP-EVs in OSA-EV microgels by incubating microgels at 37°C for 192 hours and measuring the total protein level by BCA assay. The amount of total protein in uncoated EVs decreased significantly, to 36.63% of the initial amount, whereas coated EVs retained 65.84% of their total protein **(Figure 4T).** These data revealed that OSA hydrogel had a thermal-protective effect on DP-EVs: after 192 hours of incubation, nearly twice the amount of vesicular proteins was retained by coated EVs relative to uncoated EVs.

To determine OSA-EV hydrogels steadily released DP-EVs, we incubated OSA-EV hydrogels in 3.0-μm Transwell chambers at 37°C for 192 hours. The medium was collected, and the amount of total protein was measured at predetermined time points. Before quantification of vesicular protein, vesicular membranes were destroyed using ultrasonic irradiation (521 kHz for 90 minutes). As **Figure 4U** shown**,** the concentration of released vesicular protein increased from 40.60 to 106.13 μg between 48 and 120 hours due to the steady and gentle degradation of the microgels. The cumulative amount of released vesicular protein climbed steadily in the first 48 hours, increased rapidly from 48 to 120 hours, and reached a plateau at 144 hours. Approximately 84.0% ± 4.2% of total vesicular protein was released **(Figure 4V)**.

During the hair growth cycle, the Wnt signaling pathway promotes cell proliferation and differentiation 8, MMP3 can influence adult epithelial stem cells through activation of the Wnt/β-catenin signaling pathway 45,46. MMP3 and CD9 are present in DP-EVs **(Figure 1E),** and are dynamically released from them **(Figure 4W)**. Peak release of MMP3 from OSA-EVs occurred at 96 hours, then declined to an undetectable level after 168 hours. Peak release of CD9 occurred at 48 hours, gently declined to 80.5% of the peak at 96 hours, and then declined to an undetectable level after 144 hours. These results indicate that OSA-EV hydrogels degrade over time, leading to a prolonged release of vesicular proteins. During the first 24 hours, the weight of the OSA hydrogel decreased to 80% of its initial weight, but few EVs were detected in the culture medium. The release of total vesicular protein peaked at 96 hours, when the OSA hydrogel had decreased to 31% of its initial weight, indicating that the release of EVs may rely largely on degradation of the OSA hydrogel.

### Cellular uptake of DP-EVs and proteins from OSA-EV hydrogels

**Figure 5:**
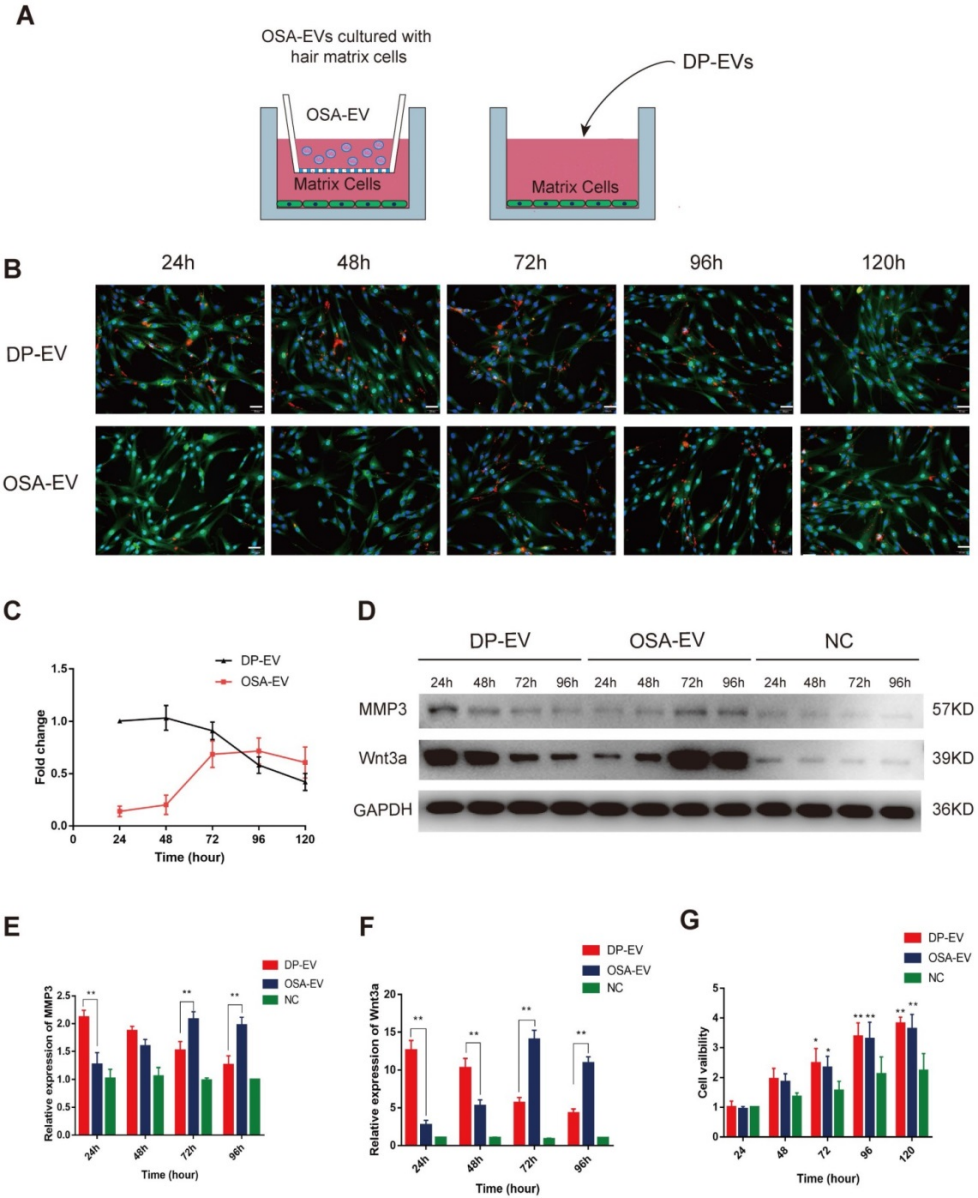
** Cellular uptake of DP-EVs and proteins from OSA-EV hydrogels.** (A) Schematics depicting hair matrix cell culture with OSA-EV hydrogel microgels and DP-EVs. The cellulose membrane of Transwell chambers has a pore size of 0.9 µm, allowing dissociated DP-EVs to pass through. Cells treated with DP-EVs were used as a control. (B) Time-lapse fluorescence images of GFP-labeled hair matrix cells taking up DIL-labeled DP-EVs from OSA-EVs or free DP-EVs. Scale bars: 50 μm. (C) Quantitative analysis of DIL signals in Figure 5B indicated retention of EVs. (D-F) After treatment with OSA-EVs and DP-EVs, the protein levels of MMP3 (D, E) and Wnt3a (D, F) in hair matrix cells were analyzed by western blotting at 24-hour intervals. (G) Treatment with OSA-EVs and DP-EVs promotes proliferation of hair matrix cells after 72 hours of incubation. Viability of cells treated with OSA-EVs and DP-EVs was significantly higher than that of cells treated with PBS. * indicates a statistically significant difference, *p* < 0.05, as compared with negative control; ** indicates a statistically significant difference (*p* < 0.01); n = 9.

To evaluate the cellular response to the dynamic degradation of OSA-EV hydrogels, we added OSA-EVs to cultured hair matrix cells. DP-EVs (40 µg) were used as a positive control, and cells treated with OSA-encapsulated DIL without EVs were used as a negative control. Before the EV uptake test, the concentration of OSA-EVs was adjusted according to the previously determined loading efficiency in order to equalize the effective vesicular concentration of OSA-EVs and DP-EVs. In brief, OSA-EV hydrogels containing 50 µg EVs were placed in a 0.9-µm Transwell chamber so that only EVs and low-molecular weight catabolites could pass through and be taken up by GFP-transfected hair matrix cells **(Figure 5A)**. Hair matrix cells treated with DP-EVs exhibited a high level of DIL staining during the first 48 hours of incubation, but at 120 hours, the DIL signal had decreased to 42.1 ± 8.2% of the initial level **(Figure 5B-C)**. Cells treated with OSA-EV hydrogel exhibited very low DIL signal during the initial 48 hours of treatment, followed by a rapid increase to 71.6 ± 12.5% at 72 hours before a slow decrease to 60.7 ± 14.9% at 120 hours **(Figure 5B-C)**. However, no DIL signals were detected during the 120-hour incubation in cells treated with OSA-encapsulated DIL without EVs. These results indicate that the OSA-EV hydrogel gradually degraded, releasing DIL-labeled EVs to the medium that were then taken up by hair matrix cells.

To further confirm that proteins released from OSA-EV hydrogel remained active and could exert a prolonged therapeutic effect, we examined protein levels of MMP3 and Wnt3a in hair matrix cells. Wnt3a, a component of the canonical Wnt signaling pathway, plays an important role in regulating follicular cell proliferation and differentiation, and ultimately influences hair regeneration and the hair cycle. Wnt3a is expressed in components of the hair follicular epithelia, especially hair matrix cells. In addition, MMP3 activates Wnt3a expression both *in vivo* and *in vitro* 45,46. As shown in **Figure 5D-F,** the levels of MMP3 and Wnt3a were relatively low in cells not treated with EVs, indicating that the increases in MMP3 and Wnt3a expression were likely due to treatment with OSA-EVs or DP-EVs. During the initial 24 hours of treatment, MMP3 protein levels were 1.7-fold higher in cells treated with DP-EVs than in cells treated with OSA-EVs, but gradually decreased over the next 48 hours, and by 96 hours had dropped to a level 1.6-fold lower than that in cells treated with OSA-EVs **(Figure 5D-E)**. Wnt3a protein levels exhibited a similar pattern: cells treated with DP-EVs had a higher level of Wnt3a than cells treated with OSA-EVs at 24 and 48 hours, but after 96 hours, their level of Wnt3a was 2.5-fold lower than that of cells treated with OSA-EVs **(Figure 5D, F)**. In cells treated with OSA-EVs, levels of MMP3 and Wnt3a peaked at 72 hours, and then gradually decreased, whereas in cells treated with DP-EVs, MMP3 and Wnt3a expression remained relatively high for the first 48 hours of incubation, and then sharply decreased. We conclude from these results that OSA hydrogel delays and prolongs vesicular protein release.

Finally, we analyzed the viability of matrix cells cultured with OSA-EVs, DP-EVs, or PBS. After 72 hours, viability was higher in hair matrix cells treated with OSA-EVs and DP-EVs **(Figure 5G)**. Because hair matrix cells are among the most important targets for DPC regulation 7,55, our system of culturing hair matrix cells with DP-EVs is an ideal model for researching signaling communication between the epithelial and mesenchymal compartments of hair follicles.

### OSA hydrogels prolong retention of DP-EVs *in vitro* hair culturing

**Figure 6:**
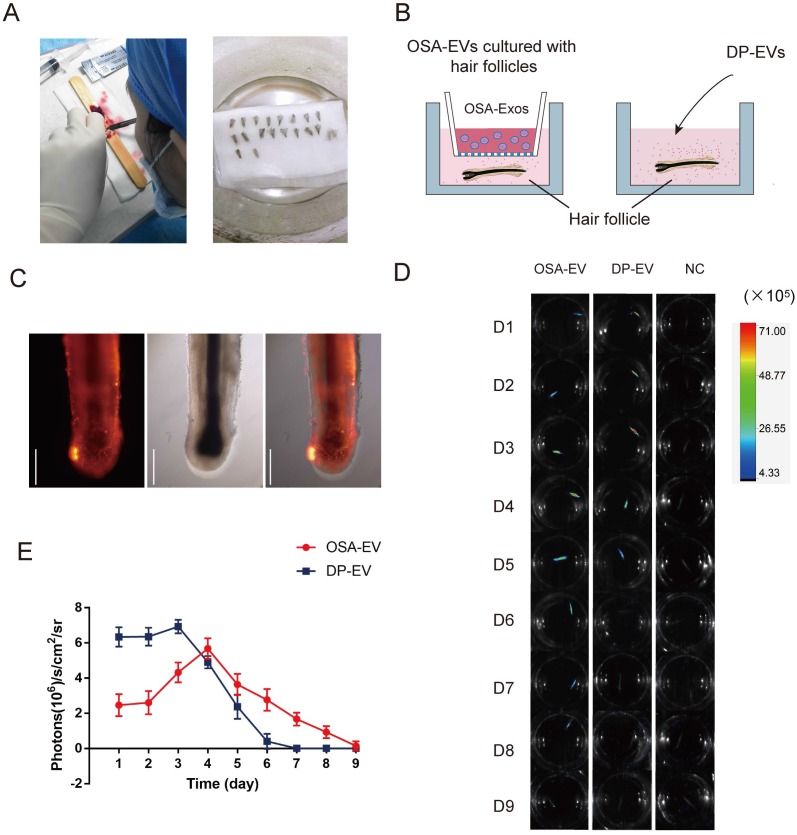
** OSA hydrogels prolong treatment of DP-EVs in *in vitro* hair cultures*.***(A) Single hair follicles were isolated and prepared from human scalp. (B) Hair follicles were cultured with OSA-EV hydrogel microgels or DP-EVs. (C) DIL labeled DP-EVs were absorbed by hair follicles. (D) *In vivo* fluorescence imaging following follicles treated with DIL labeled OSA-EV, DP-EV, or DIL without EVs (as a negative control). (E) Quantitative analysis of DIL signal intensity, expressed as photons/s/cm^2^/sr. Data were expressed as means ± s.d. n =15.

Hair follicles were collected and isolated from healthy female adults (5 woman aged between 35 and 45 yr) after informed consent was obtained from all donors. Follicle isolation and culture were performed as described previously 56,57 **(Figure 6A)**.

To determine whether DP-EVs could gradually escape from OSA-EV hydrogels and be absorbed by hair follicles, we used encapsulated and non-encapsulated DP-EVs **(Figure 6B)** to interfere with cultured hair follicles *in vitro*, and then used an *in vivo* imaging system to detect the duration of DIL-labeled EVs in hair follicles. Follicles were imaged at 24-hour intervals for 9 days **(Figure 6D)**. After 24 hours of culture, DIL-labeled DP-EVs were absorbed by hair follicles, as determined by fluorescence microscopy **(Figure 6C, S4)**. However, no DIL-positive signal was detected in follicles cultures with DIL solution without EVs for the same period **(Figure 6D, S5)**. Thus, only when bioactive EVs were absorbed by the hair follicles could the signal could be detected. In the DP-EV treatment group, the signals diminished after 5 days, whereas in the OSA-EV-treated group, DIL signals were still detectable after 8 days of culture before they diminished **(Figure 6D)**. Fluorescence intensity analysis revealed that DIL signals in hair follicles from the OSA-EV group decreased more gradually and lasted longer **(Figure 6E)**. This phenomenon indicated that OSA hydrogel gradually released DP-EVs and protected them from degradation *in vitro*, which in turn allowed the EVs to persist for a longer period of time in the culture medium. In the end, OSA hydrogel helped follicles fully uptake DP-EVs.

### OSA hydrogels increased the therapeutic effect of DP-EVs on human hair follicle growth in organ culture

Human hair growth *ex vivo* is correlated with *in vivo* hair growth, so we used an ex vivo organ culture model to study the effect of EVs on DP-EV-treated human HFs. In hair follicle organ culture experiments, change in hair growth is commonly used to measure follicular activity, anagen hair follicles will continue to grow for several days, maintaining the anagen morphology and epithelial-mesenchymal interactions.

**Figure 7:**
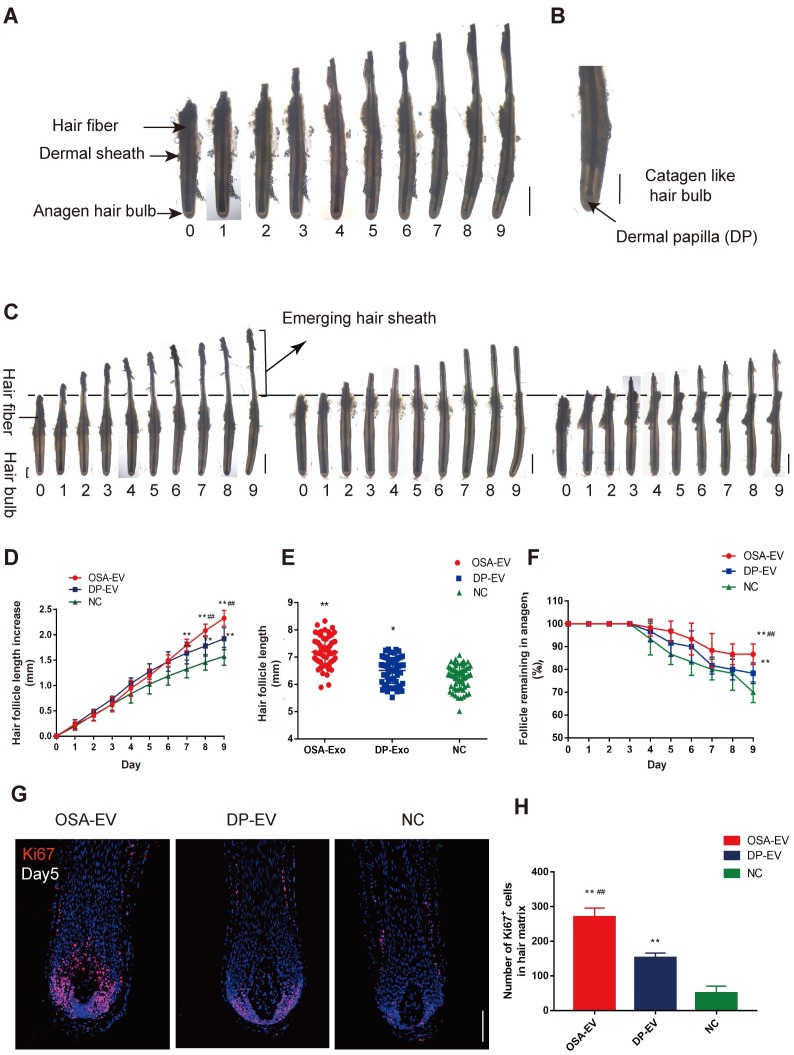
** OSA hydrogels increased the therapeutic effect of DP-EVs on human scalp hair follicle growth in organ culture.** (A) Sequential photomicrographs, taken every 24 h for 9 d, of individual scalp follicles in organ culture under various conditions. Images show growth of hair fiber and inner and outer root sheath, but not dermal sheath. Scale bars: 1 mm. (B) By day 8, some hair follicles exhibited a catagen-like morphology: pigmentation had ceased, and the dermal papilla (DP) was detached from hair fiber and hair matrix. Scale bars: 0.5 mm (C) Hair follicles were cultured in OSA-EVs (containing 300 μg DP-EVs in 300 µl culture medium), 300 μg DP-EVs , or negative control medium. Scale bars: 1 mm. (D-F) Hair follicles in each group were assessed and measured daily for increase in hair length (D), hair length (E), and the percentage of hair follicles remaining in anagen (F). (G) Proliferating cell nuclear antigen (Ki67, red) was detected by immunofluorescence analysis in hair matrix at day 5 in each group. Scale bars: 50 µm. (H) Quantitative analysis of the number of Ki67^+^ cells in hair matrix. Data are expressed as mean ± s.d. Five individuals were used per experiment and at least 12 follicles/person were examined for each condition. *, p < 0.05 relative to the negative control (NC); **, p < 0.01; #, p < 0.05 relative to DP-EVs; ##, p < 0.01. Statistical significance was determined by ANOVA with Bonferroni correction.

To determine whether DP-EVs could affect scalp hair follicle growth, we grew isolated hair follicles in basic culture medium supplemented with different concentrations of DP-EVs and collected daily images for assessment of their morphology and physical parameters. Sequential photomicrographs were taken every 24 h for 9 d of individual scalp follicles in organ culture under various conditions. The negative control exhibited growth of the hair fiber and inner and outer root sheath, but not the dermal sheath **(Figure 7A)**. By day 8, some hair follicles exhibited a catagen-like morphology: pigmentation had ceased, and the dermal papilla (DP) was detached from hair fiber and hair matrix **(Figure 7B)**. Hair length, the increase in hair follicle length, and the number of follicles remaining in anagen were recorded each day, and the data showed that DP-EVs stimulated the growth of human hair follicles in a concentration-dependent manner **(Figure S6)**. Hair follicles treated with 1 μg/μl DP-EVs had a significantly stronger effect on stimulating hair follicle growth than lower concentrations (0.1 μg/μl and 0.01 μg/μl). We compared the therapeutic effect of DP-EVs with that of DF-EVs, KC-EVs and PBS **(Figure S7-S8)**. The results showed that DP-EVs had a significantly stronger effect on stimulating hair growth and prolonged anagen than both DF-EVs and KC-EVs. At day9, the hair length increment was 1.35- and 1.25-fold higher than that in the DF-EV-treated group and KC-treated group, respectively **(Figure S7B)**. And the rate of hairs in anagen in DP-EV-treated group was ~1.2 higher than other groups **(Figure S7C)**. DF-EVs and KC-EVs showed no significant effect on stimulating hair growth and extending HF anagen than NC (**Figure S7A-C**). In line with this, only DP-EV-treated hair follicles showed significant larger (p<0.01) number of Ki67-pisitive cells compared with NC in hair matrix on day 5 (*p*<0.01) **(Figure S8)**.

To assess if OSA hydrogel improved the therapeutic effect of DP-EVs in organ culture, we added OSA-EVs (containing equal amount of DP-EVs) to cultured human hair follicles. As a positive control we used 1 µg/µl DP-EVs, and as a negative control we used hair follicles treated with OSA without EVs **(Figure 7C)**. The results revealed that OSA-EVs and DP-EVs stimulated human hair follicles to grow faster in organ culture **(Figure 7C-E)**. By day 9, the length of hair follicles cultured with OSA-EVs increased 2.32 ± 0.16 mm, whereas follicles cultured with DP-EVs or PBS increased 1.93 ± 0.22 mm and 1.58 ± 0.18 mm, respectively **(Figure 7D).** The total follicular length in the OSA-EV-treated group at day 9 was 7.20 ± 0.54mm, 1.11- and 1.15-fold higher than that in the DP-EV-treated group and negative control, respectively **(Figure 7E)**. Addition of OSA-EVs and DP-EVs to culture medium also increased the number of hair follicles in anagen **(Figure 7F).** Anagen on day 9 was prolonged by ~16.67% with OSA-EVs and ~8.33% with DP-EVs. We also assessed cell viability of human hair follicles based on Ki67 immunofluorescence, and found that the OSA-EV treatment group had the most Ki67-positive cells in the hair matrix on day 5 (P<0.01) **(Figure 7G-H),** indicating that hair matrix in the OSA-EV-treated group was most active.

In contrast to natural HFs *in vivo*, anagen HFs cultured *in vitro* quickly undergo catagen development due to trauma. Most *ex vivo* HFs in normal William's E medium begin to enter catagen and stop growth at day 9, as described previously 57. Hair elongation and the rate of anagen maintenance reflect differences in the anagen-to-catagen period because when HFs stop growth, they begin to grow inactive and die 57,58. Together, these data indicated that OSA hydrogels increased the therapeutic effect of DP-EVs on cultured human hair follicles, thereby accelerating hair fiber growth and prolonging anagen via dramatic activation of the hair matrix.

### OSA hydrogels improve retention of DP-EVs *in vivo*

**Figure 8:**
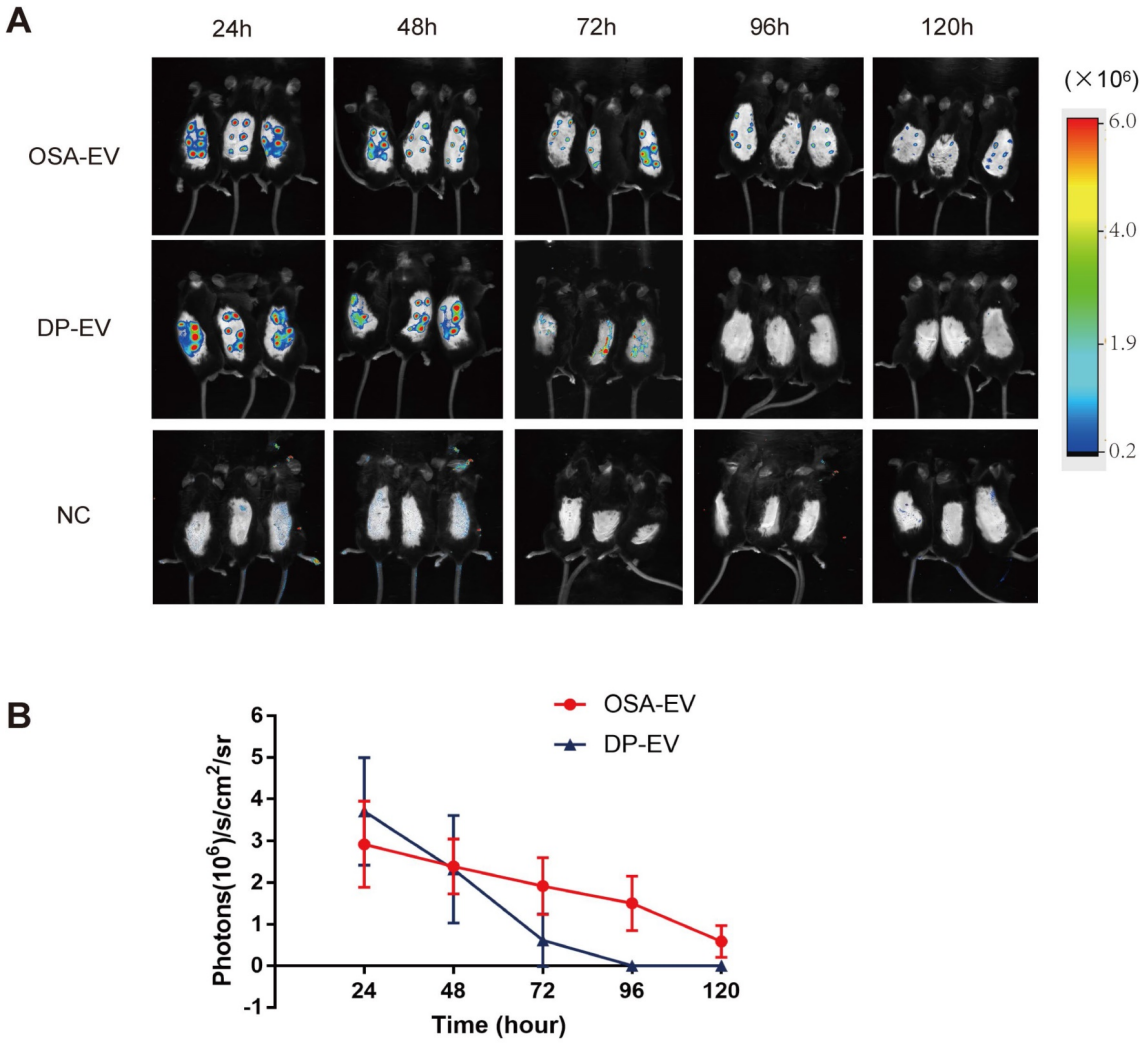
** OSA hydrogel improved the stability and retention of DP-EVs and enhanced the hair-promoting effect.** (A) *In vivo* fluorescence imaging following subcutaneous injection of DIL-labeled OSA-EVs (containing 5 mg EVs per mouse), DIL-labeled DP-EVs (4 mg EVs per mouse), and PBS. (B) Quantitative analysis of the DIL signal intensity, expressed as photons/s/cm^2^/sr. Data are expressed as means ± S.D.; n = 32 (eight mice per group).*, *p* < 0.01 relative to negative control; ^#^, *p* < 0.01 relative to minoxidil; ^+^, *p* < 0.01 relative to DP-EVs. Statistical significance was determined by one-way ANOVA with Bonferroni correction.

To determine whether OSA hydrogels improve the retention of DP-EVs *in vivo*, mice subjected to dorsal hair depilation were subcutaneously injected with DIL-labeled OSA-EVs and DP-EVs; OSA lacking EVs were used as a negative control. The mice were imaged using an IVIS fluorescence imaging system at 24-hour intervals. Twenty-four hours post-treatment, strong fluorescence signals were observed on the backs of mice treated with OSA-EVs and DP-EVs. In the DP-EV-treated group, the signals diminished after 48 hours, and decreased to a nearly undetectable level at 72 hours. This phenomenon was also observed in a previous study in which MSC-derived EVs were subcutaneously injected 29. By contrast, in the OSA-EV-treated group, DIL signals were visible until 96 hours post-injection, becoming nearly undetectable only after 120 hours **(Figure 8A-B)**. Fluorescence intensity analysis revealed that DIL signals from OSA-encapsulated EVs decreased more gradually and lasted longer **(Figure 8B)**. IVIS fluorescence imaging revealed that the vesicular signals in mice treated with OSA-EV hydrogels were present for 2 days longer than in mice treated with uncoated EVs. Although OSA-EVs degraded sufficiently slowly to continue to release EVs for 7 days, we are concerned that body movement, body fluid, and blood flow might accelerate OSA hydrogel degradation.

### OSA hydrogels accelerate the therapeutic effects of DP-EVs on the telogen-to-anagen transition

At first, we investigated the therapeutic effect of DP-EVs *in vivo*, DF-EVs and KC-EVs were applied as control (**Figure S9-10**). For this purpose, we used C57BL/6 mice for treatment by depilating a patch of dorsal hair. The results revealed that DP-EVs were the most effective at accelerating the hair transition from the telogen to anagen phase **(Figure S9)**. The hair coverage rate at day18 was ~83% in the DP-EV-treated group, which was about 1.76- and 2.26- fold higher than DF-EV-treated and KC-EV-treated group. The hair coverage rate was no significant difference in mice treated with DF-EVs, KC-EVs, and PBS at day 18. Moreover, DP-EVs treatment significantly increased the number of Ki67-pisitive cells (P<0.01) in hair matrix on day 18 **(Figure S10)**.

**Figure 9:**
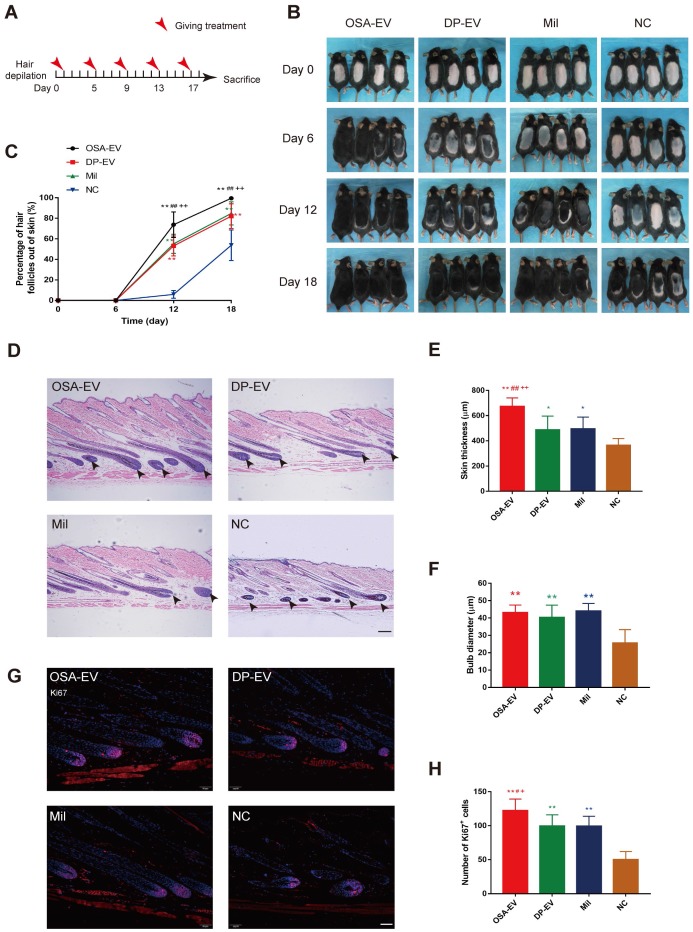
** OSA-EVs and DP-EVs accelerate the telogen-to-anagen transition in C57BL/6 mice.** (A) After hair depilation, mice dorsal skin was treated every 4 days with OSA-EVs, DP-EVs, PBS (negative control), or 3% minoxidil (positive control). Observation continued over an 18-day treatment period, then animals were sacrificed for further analysis. (B) The dorsal skin was photographed at 0, 6, 12, and 18 days. (C) Areas with hair regrowth out of skin were quantified using Image-Pro Plus software. (D) HE-stained sections of dorsal skin from the OSA-EVs treatment groups at day 21 displayed longer and larger HFs than those from the DP-EVs treated, minoxidil-treated and PBS-treated groups (arrow: dermal papilla). Scale bars: 50 µm. (D, E) Anagen HFs in the OSA-EV-treated were advanced, with a significant increase in skin thickness (E) relative to the DP-EV-, minoxidil-, and PBS-treated groups. (F) Hair bulbs of mice treated with OSA-EVs were of larger diameter than those of the PBS-treated group, and not significantly different from those of the DP-EVs treatment group and minoxidil-treated group. (G) Images of HFs at day 21. Ki67^+^ cells (red) were located in the hair matrix and DP compartments. Nuclei are stained with DAPI (blue). Scale bars: 50 µm. (H) Ki67 expression on day 21 was highest in the OSA-EV-treated group. Ki67 expression was higher in mice treated with DP-EVs or minoxidil than in the control group, and there was no difference between the DP-EVs and minoxidil groups. Each bar represents the mean ± S.D. of eight replicates. *, *p* < 0.05 relative to the negative control; **, *p* < 0.01 relative to the negative control; ^#^, *p* < 0.05 relative to minoxidil; ^##^, *p* < 0.01 relative to minoxidil; ^+^, *p* < 0.05 relative to DP-EVs; ^++^, *p* < 0.01 relative to DP-EVs. Statistical significance was determined by one-way ANOVA with Bonferroni correction; n = 8 for each group.

Next, we sought to confirm that OSA hydrogels promote the therapeutic effects of DP-EVs on hair regrowth. Hair growth in groups treated with DP-EVs and OSA-EVs was compared with hair growth in mice externally treated with 3% minoxidil, currently considered to be the gold standard for hair regrowth treatment 59, as well as in mice treated with PBS (as a negative control). Treatment intervals were determined based on the length of time that OSA-EVs were retained in the recipient area, as shown in **Figure 8**. We performed treatment with OSA-EVs, DP-EVs, minoxidil, or PBS treatment every 4 days **(Figure 9A)**.

Depilated skin of C57BL/6 mice is pink during telogen phase and darkens with anagen initiation 60. Most mice in the four groups initiated darkening after 12 days, indicating the onset of anagen phase **(Figure 9B)**. The depilated area of mice in the OSA-EV-treated groups darkened and exhibited more than 70% hair regrowth by 12 days after initiation of treatment, and almost full hair regrowth by day 18. The minoxidil and DP-EV-treated group exhibited approximately 84% hair regrowth at 18 days. By contrast, the control group exhibited about 6% hair regrowth at day 12, and only 54% hair regrowth was observed at the final time point **(Figure 9C)**. These data revealed that OSA-EVs were the most effective at accelerating the hair transition from the telogen to anagen phase.

Patches of dorsal skin were fixed for HE staining after 18 days of treatment **(Figure 9D)**. Hair follicles that had been treated with OSA-EVs, DP-EVs, and minoxidil exhibited typical features of anagen, such as thicker skin and a larger hair bulb. Skin was thickest in the OSA-EV-treated group **(Figure 9E)**, whereas there were no significant differences in bulb diameter among the treatment groups **(Figure 9F)**. Sections from dorsal skin of mice injected with DIL-labeled OSA-EV hydrogels or EVs revealed DIL staining in hair bulbs and dermal shafts after 3 days of treatment (**Figure S11, arrows**), indicating that hair follicle cells, including hair matrix cells, took up EVs *in vivo*.

Furthermore, we assessed cell viability in hair follicles by Ki67 immunofluorescence; Ki67 was expressed in cell nuclei, mostly in hair matrix cells **(Figure 9G)**. The OSA-EV-treated had the greatest number of Ki67-positive cells **(Figure 9H)**, followed by the DP-EV- and minoxidil-treated groups. There was no significant difference between the DP-EV and minoxidil groups. These data indicated that OSA *in vivo* could enhance the retention and stability of DP-EVs, further accelerate hair matrix proliferation, and promote telogen-to-anagen transition of hair follicles.

### EV therapy alters expression of signaling molecules in hair follicles

**Figure 10:**
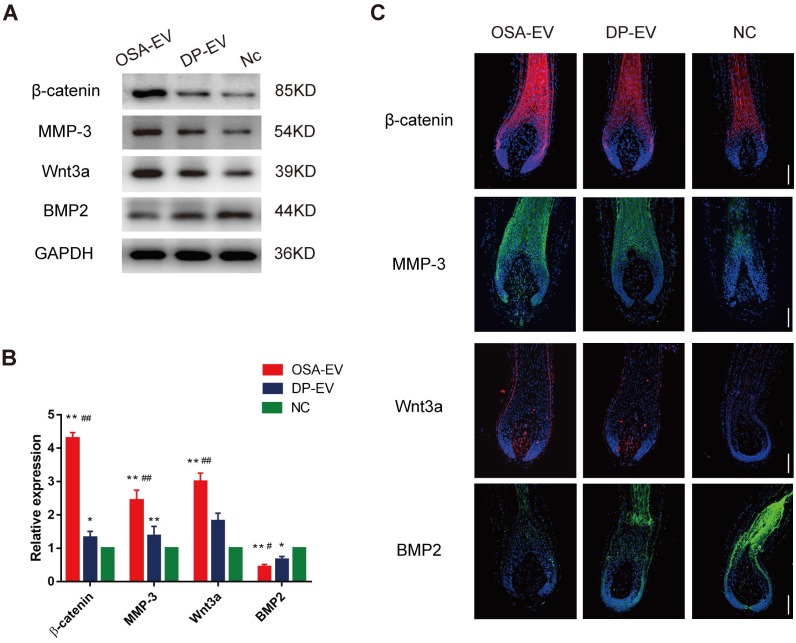
** Western-blot and immunofluorescence analysis of cultured hair follicles.** (A-B) Western-blot analysis was performed 5 days after treatment. The relative levels of β-catenin, Wnt3a, MMP3 and BMP2 of cultured hair follicles treated with OSA-EVs, DP-EVs or PBS by day 5. (C) β-catenin was detected by immunofluorescence at day 5, and was present throughout the hair follicle, except for the dermal papilla. Fluorescence intensity was strongest in the OSA-EV-treated group followed by the DP-EV-treated group. MMP3 typically localized to the DP and hair root sheath. MMP3 was detected at high levels in the hair follicles of the OSA-EV-treated group, and low levels in the DP-EV-treated group; the lowest MMP3 level was observed in the hair follicles in the negative control. Similar to MMP3, Wnt3a was detected at DP and hair root sheath. The fluorescence intensity was much stronger in the OSA-EV-treated groups than in the other two groups. BMP2 expression was very low in the epithelial compartment of hair follicles in the OSA-EV-treated groups, but was upregulated in the DP-EV group and highest in the PBS-treated group. Scale bars: 100 µm. *, p < 0.05 relative to the negative control; #, p < 0.05 relative to minoxidil treatment; +, p < 0.05 relative to DP-EV treatment; ++, p < 0.01 relative to DP-EV treatment, Statistical significance was determined by one-way ANOVA with Bonferroni correction; n = 5 for each group.

To elucidate the mechanism underlying promotion of hair growth by DP-EVs, we analyzed the signaling molecule changes both *in vitro* and *in vivo* experiments. For the *in vitro* experiments, we chose anagen hair follicles from each group at day 5, when EV absorption could still be detected in both OSA-EV- and DP-EV-treated hair follicles in the middle-phase of treatment, and subjected them to histological analysis **(Figure 6D)**. In a previous study, western blots revealed high levels of MMP3 in vesicular protein** (Figure 1E)**, which activated Wnt signaling pathways as reflected by Wnt3a expression 45,46; this was confirmed in our experiments for this study **(Figure 5D)**. Wnt and BMP signaling pathways play important roles in hair regeneration and the hair cycle transition. For example, Wnt signaling molecules such as Wnt3a boost proliferation of follicular precursor cells proliferation, thereby promoting promotes the telogen-to-anagen transition 61,62. However, BMP signaling molecules such as BMP2 and BMP4 maintain the resting state of stem cells, thereby inhibiting activation of follicular precursor cells 63,64. To confirm the key signaling protein changes in HFs treated with DP-EVs treatment *in vitro*, we performed western blotting and immunofluorescence to detect MMP3, β-catenin, Wnt3a, and BMP2. Western blotting revealed that the levels of Wnt3a, MMP3, and β-catenin were significantly raised in cultured HFs treated with OSA-EVs and DP-EVs. By day 5, the levels of Wnt3a, MMP3 and β-catenin in OSA-EV-treated group were 3.0- (P<0.01), 4.3- (P<0.01), and 2.4-fold (P<0.01) higher than in the negative control. By contrast, the expression levels in follicles cultured with DP-EV increased only 1.8- (P<0.01), 1.3- (P<0.05), and 1.4-fold, respectively **(Figure 10A-B)**. However, expression of BMP2, which inhibits hair growth and anagen maintenance, was dramatically reduced in the OSA-EV-and DP-EV-treated groups, decreasing by 0.4- and 0.6- fold (P<0.05), respectively, relative to the negative control **(Figure 10A-B)**. This tendency was also confirmed by immunofluorescence staining: staining for β-catenin, MMP3, and Wnt3a was strongest in follicles treated with OSA-EVs, followed by follicles treated with DP-EVs. By contrast, BMP2 staining was weakest in follicles treated with OSA-EVs, and second weakest in follicles treated with DP-EVs **(Figure 10C)**.

**Figure 11:**
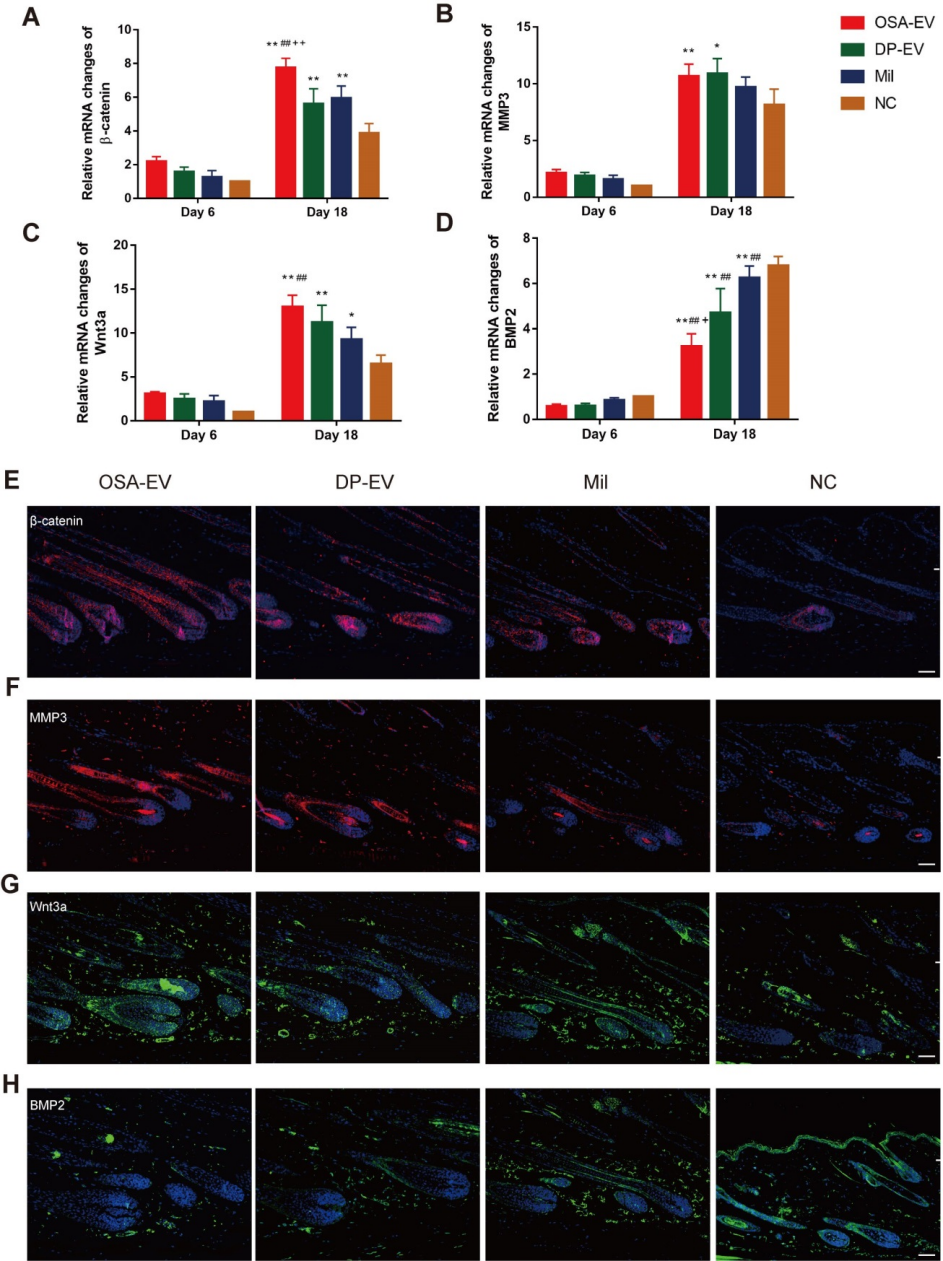
** qRT-PCR and immunofluorescent analysis of hair follicle regeneration.** (A-D) qRT-PCR analysis was performed 6 and 18 days after treatment. Treatment did not result in changes in mRNA expression at day 6. By day 18, the OSA-EV-treated group expressed the highest levels of β-catenin (A), MMP3 (B), and Wnt3a (C), and the PBS-treated group expressed the lowest levels of these proteins. By contrast, the OSA-EV-treated groups expressed the lowest levels of BMP2 (D), and the PBS-treated group expressed the highest level of this protein. (E) β-catenin was present throughout the hair follicle at day 18, as revealed by immunofluorescence. Fluorescence intensity was strongest in the OSA-EV-treated group, followed by the DP-EV-and minoxidil-treated groups. (F) MMP3 typically localized to the DP and hair root sheath. MMP3 was detected at high levels in the hair follicles of the OSA-EV-treated groups, and at lower levels in the DP-EV-and minoxidil-treated groups; it was almost undetectable in the PBS-treated group. (G) Wnt3a was detected in DP and hair root sheath, similar to MMP3. The fluorescence intensity was much stronger in the OSA-EV-treated groups than in the other three groups. (H) BMP2 expression was very low in the epithelial compartment of hair follicles in the OSA-EV-treated group, but was upregulated in the DP-EV-treated group and minoxidil group and highest in the PBS-treated group. Scale bars: 50 µm. *, *p* < 0.05 relative to the negative control; ^#^, *p* < 0.05 relative to minoxidil treatment; ^+^, *p* < 0.05 relative to DP-EV treatment; ^++^, *p* < 0.01 relative to DP-EV treatment. Statistical significance was determined by one-way ANOVA with Bonferroni correction; n = 8 for each group.

As in the *in vivo* experiments, we analyzed changes in signaling molecules in the early and late process of treatment by RT-PCR. We chose day 6 and day 18 as observation time points because all mouse HFs were still in telogen at day 6, but had entered into anagen phase at day 18 **(Figure 11D)**. As shown in **Figure 11A-D**, expression of mRNAs encoding signaling molecules increased from late telogen to mid-anagen phase. Treatment with OSA-EVs, DP-EVs, or minoxidil increased gene expression of MMP3 and Wnt signals in hair follicles at day 18 **(Figure 11A-D)**, but no significant changes were observed on day 6 **(Figure 11A-D)**. With OSA-EV treatment, the levels of β-catenin, Wnt3a, and MMP3 increased 2.0-, 1.7-, and 1.3-fold, respectively, relative to the control. A similar trend was observed in the DP-EV- and minoxidil-treated groups, but the increases were smaller. By contrast, treatment with OSA-EVs, DP-EVs, or minoxidil decreased expression of BMP signaling pathway components **(Figure 11D):** a 0.5-, 0.7-, and 0.9-fold reduction in BMP2 expression following treatment with OSA-EVs, DP-EVs, and minoxidil, respectively.

To further investigate the signaling changes caused by EV treatment, we performed hair follicles immunofluorescence staining for β-catenin, MMP3, Wnt3a, and BMP2 at day 18, by which time HFs of all groups entered into anagen phase. We observed the strongest staining of β-catenin, MMP3, and Wnt3a in mice treated with OSA-EVs, followed by those treated with DP-EVs and minoxidil** (Figure 11E-G)**. However, the opposite trend was observed in BMP protein expression among the four groups: mice treated with OSA-EVs expressed the lowest levels of BMP2. These results indicate that EV treatment altered both mRNA and protein levels of signaling molecules, increasing the expression of activators and repressing inhibitors of hair growth. Previous study have demonstrated that dermal fibroblasts-derived EVs did not promote hair growth in cultured human hair follicles unless stimulated by bFGF and PDGF-AA 33,34. We speculate that the reason for this may be the specific contents of DP-EVs carrying different kinds of regulatory proteins, mRNAs, and micro-RNAs promote hair growth through mechanism involves hair growth-promoting signaling pathways such as MMP3 regulated Wnt-β-catenin signaling pathway 8,45,46, along with downregulation of inhibitory signaling pathways such as BMP signaling pathway 64. Of interest, a recent study showed that stimulated DF-EVs activate indirectly beta-catenin pathway via secretion of norrin by DP 34. These results revealed that the signal communication between DPCs and hair matrix cells may not be irreplaceable during the process of hair growth.

## Conclusions

In summary, we successfully isolated EVs from the culture medium of low-passage hDPCs and confirmed that DP-EVs contribute to the proliferation of hair matrix cells, as well as hair regrowth. Furthermore, we encapsulated DP-EVs in sodium alginate with a 5% DO to form degradable and injectable OSA-EV microgels. These OSA hydrogels degraded over time to release DP-EVs, which could then be absorbed by hair matrix cells and hair follicles, resulting in improved cell viability and accelerated hair follicle regeneration. OSA increased the retention and stability of DP-EVs and further promoted the therapeutic effects for hair regrowth. The mechanism underlying the therapeutic effect of EV treatment likely involves upregulation of hair growth-promoting signaling molecules such as MMP3, Wnt3a, β-catenin, along with simultaneous downregulation of inhibitory signals such as BMP2 **(Scheme 1)**. This research had some limitations: for example, we need to further explore the biological mechanism underlying the effect of DP-EVs on hair growth, and vesicular proteins other than MMP3 should be studied in greater detail. In the future, it will be beneficial to find biomaterials with more controllable degradation, which could be used for encapsulation to gently release EVs and protect their bioactivity. Our results represent an important first step in exploring alternative treatments for hair loss using DP-EVs and engineered EVs, and it could be a great improvement for the long term therapeutic use of EVs.

## Methods

*Preparation and characterization of OSA:* Sodium alginate (10 g; Sigma-Aldrich, Milwaukee, WI, USA) was dissolved in 100 ml distilled water/ethyl alcohol (1:1, v/v), and oxidized with sodium periodate as previously reported 48. The ratios of guluronate and periodate used were 100:2.5, 100:5, 100:7.5, 100:10, 100:20, and 100:30. After a 4 hour incubation in the dark, the reaction was quenched by adding an equimolar amount of ethylene glycol for 30 minutes. After filtering, the resulting solution was washed with ethanol/water (1:1, v/v) and freeze-dried.

*Degradation kinetics assay:* Native alginate and OSA were dissolved in PBS (pH 7.4) to obtain a 0.5% (w/v) solution, then incubated with 5% CO_2_ at 37 °C. At appropriate time intervals, the viscosity of the solution was measured using an Ubbelohde viscometer. The percent degradation was calculated using the following equation:

Degradation (%) = M_t_/M_0_ × 100

where M_t_ and M_0_ are the viscosity-average molecular weights at time t and time 0, respectively.

*Preparation of hydrogels:* Alginate and OSA with varying DOs (2.5%, 5%, 7.5%, 10%, and 20%) were dissolved in distilled water to obtain 1.5%, 1.5%, 1.5%, 1.5%, 2.5%, and 3.5% (w/v) solutions, respectively, and sterilized by filtration. Native alginate and OSA solutions were dropped into 0.1 M CaCl_2_ using a microinjection Femtotip (40 µm inner diameter; Eppendorf TransferMan NK2, Hamburg, Germany), and incubated for 15 minutes to initiate gelation. After gelation, the hydrogels were washed three times with PBS.

*Swelling and degradation kinetics of hydrogels:* Native alginate and OSA hydrogels were washed three times with PBS, and dried at 70 °C until constant mass was achieved. The dry weights (W_i_) of the fabricated films were then measured using an electronic microbalance (Mettler-Toledo, Greenville, SC, USA). Dried hydrogel samples were immersed in PBS at 37 °C. At predetermined time points, samples were removed and washed with PBS. The excess surface solution was removed by filter paper, and the swollen hydrogel sample weights (W_t_) were measured. Fresh culture medium was then added to the samples. The swelling ratio (SR) was calculated using the following equation:

SR (%) = (W_t_-W_i_)/W_i_ × 100

where W_t_ and W_i_ represent the weights of the swollen and dried samples, respectively.

For analysis of degradation kinetics, microgels were weighed prior to incubation in PBS (W_0_) then weighed again at different time points, after removal of surface medium (W_d_). Weight loss (%) was calculated according to the following equation:

weight loss (%) = (W_0_-W_d_)/W_0_ × 100

where W_0_ and W_d_ are the weights of samples before and after degradation, respectively.

*Cell culture:* Healthy human scalp specimens were obtained from patients undergoing facelift surgery, with informed consent and the approval of the Plastic and Cosmetic Surgery Nanfang Hospital of Southern Medical University (Guangzhou, China). Human hair papilla cells (hDPs) were isolated as described previously 15,16,39 and cultured in DMEM (Gibco, Grand Island, NY, USA) with 10% exosome-free FBS (CellMax, Beijing, CN, Cat. #CMS101.03) and 100 U/ml penicillin-streptomycin (ScienCell, San Diego, CA. USA. Cat. No. 0503). Human hair matrix cells (ScienCell, San Diego, CA. USA. Cat. #2410), dermal fibroblasts (ScienCell, Cat. #2320) and epidermal keratinocytes (ScienCell, Cat. #2110) were all purchased from from ScienCell Research Laboratories (San Diego, San Diego, CA. USA). As is recommended by ScienCell Research Laboratories, hair matrix cells were cultured in MSC medium (ScienCell, Cat. #7501) with 5% exosome-free FBS, 1% stem cell growth supplement (ScienCell, Cat. #7562) and 1% penicillin-streptomycin. Keratinocytes were cultured in keratinocyte Medium (ScienCell, Cat. #2101) with 1% keratinocyte growth supplement (ScienCell, Cat. No. 2152) and 1% penicillin/streptomycin solution (ScienCell, Cat. No. 0503). Dermal fibroblasts were cultured in DMEM supplemented with 10% exosome-free FBS. Cells were incubated with 5% CO2 at 37 °C.

*Isolation of DP derived EVs:* Media were collected from low-passage hDPC cultures (P1-P3) and stored at -20 °C. EVs were isolated using ultracentrifugation (SW 32 Ti swinging-bucket rotor in a Beckman Coulter L8-80M Ultracentrifuge), as described previously 29. Briefly, 500 ml hDPC culture medium was centrifuged at 2000 × *g* for 20 minutes, then at 10,000 × *g* for 20 minutes, to remove cells and debris. The supernatant was filtered through a 0.2 µm syringe filter (Corning, Germany), and ultracentrifuged at 100,000 × *g* for 60 minutes at 4 °C twice. The final pellet was suspended in 100-200 µl PBS and stored at ^-^80 °C. Dermal dermal fibroblasts drived EVs (DF-EVs) and keratinocyte derived EVs (KC-EVs) were isolated from low-passage (P1-P3) dermal fibroblasts keratinocytes cultures, which were prepared according to the steps described above. As a negative control, an equal volume of 1× PBS was used, which was prepared in the same way as the actual EVs samples.

*Nanoparticle tracking analysis (NTA):* The particle size of hDP-EVs was investigated by nanoparticle tracking analysis (NTA) with a NanoSight NS300 (Malvern Panalytical, UK). Before acquisition, the samples were diluted in Milli-Q water (1:1000) due to their high concentration. Three individual measurements were obtained and analyzed by NTA version 2.3 software (NanoSight Ltd, Wiltshire, UK).

*Transmission electron microscopy (TEM):* A pellet of EVs was resuspended in PBS at a concentration of 1 mg/ml. Five microliters of this EV solution was added to Formvar/Carbon transmission electron microscopy (TEM) grids, then covered with absorbent membranes and air-dried at room temperature for 20 minutes. Grids were washed with PBS, stained with 3% (w/v) phosphotungstic acid (Electron Microscopy Sciences, Washington, PA, USA), and air-dried at room temperature. The grids were then washed three times with distilled water and examined with a Hitachi H-600 transmission electron microscope (Hitachi, Tokyo, Japan) at 100 kV.

*Scanning electron microscopy (SEM):* OSA-EVs were fixed in 2% EM grade glutaraldehyde (Electron Microscopy Sciences) for 90 minutes. Post-fixation, sample seeded films were dehydrated in a graded ethanol series (30%, 50%, 70%, 80%, 90%, 95%, and 99.8% v/v), then dried with 5 ml hexamethyldisilazane (HMDS) for 5 minutes. Filters were removed, air-dried, and mounted on aluminum stubs and sputter coated with gold before SEM examination (HITACHI X-650).

*Western blotting:* Protein extracts were isolated from EVs using RIPA protein lysis buffer containing 1 mM PMSF. The protein concentration in EVs was determined by using a BCA protein assay kit (Pierce, Rockford, IL, USA) and measuring absorbance at 562 nm. Total protein (20 µg) was subjected to SDS‑PAGE and transferred to a polyvinylidene fluoride membrane. The membrane was blocked in 5% BSA for 1 hour, and probed with appropriate primary antibodies overnight at 4 °C. Primary antibodies against the following proteins were used: MMP3 and Wnt3a (1:1000 dilution; Abcam, Cambridge, UK); Calnexin, CD9, and ALIX (1:800 dilution; Abcam); GAPDH (1:1000 dilution; Cell Signaling, Beverly, MA, USA). Blots were incubated with goat anti-rabbit IgG (H+L) (1:1000 dilution; Abcam) for 2 hours, then washed with PBS. Immunoreactive bands were detected with a chemiluminescent substrate (ECL TransGen Biotech, China). For the cellular protein assay, hair matrix cells were lysed and subjected to western blot analysis as described previously 35,65. Antibodies were used as described above.

*EV labeling and uptake assay:* The precipitate of EVs with 100 µl supernatant was labeled using the DIL Fluorescent Cell Linker Kit (1 µl DIL for 100 µl solution ) for General Cell Membrane Labeling (Thermo Fisher Scientific, Waltham, MA, USA) without suspension, after a 20-minute incubation at room temperature, the supernatant was removed. The precipitate was washed with 50ml fresh PBS one time, followed by filling new fresh PBS to resuspend precipitate. The sample was ultracentrifugation at 100,000 × g for 1 hour twice to wash the dye. As a negative control, an equal volume of PBS supernatant was mixed with DIL, and then was prepared in the same way as the actual exosome samples. Hair matrix cells were cultured in a 24-well plate and transfected with GFP slow virus (Sigma-Aldrich) according to the manufacturer's instructions. For the matrix cell uptake assay, an appropriate number of EVs, as determined by BCA protein assay, was added to the cell culture. Hair matrix cells were cultured with EVs for 24 hours, and their interaction was evaluated by confocal laser scanning microscopy (LSM510; Carl Zeiss, Jena, Germany).

*In vitro cell viability assay:* For cell proliferation assays, 5 × 10^3^ hair matrix cells were seeded into 96-well plates, and treated with 0.08 µg/µl hDP or 0.08 µg/µl OSA-EVs. OSA hydrogel served as a negative control. OSA-EVs and OSA were placed in 0.9 μm Transwell chambers to control for the influence of OSA fragments. Proliferation and cell viability were measured every 24 hours using a Cell Counting Kit-8 (CCK-8; Dojindo, Japan) according to the manufacturer's instructions. The absorbance was read at 490 nm on an enzyme-linked immunosorbent assay (ELISA) reader (BioTek, Vermont, VT, USA). All assays were performed using biological and technical triplicates.

*Cell cycle assay:* The cell cycle in hair matrix cells cycle was analyzed by flow cytometry. After incubation with different concentration of DP-EVs , DF-EVs, KC-EVs or PBS for 48 h, cells were harvested with 0.05% trypsin and washed with PBS. Samples were stained with propidium iodide (PI)-RNase (BD Biosciences, San Jose, CA, USA). All samples were analyzed on a FACSCanto II Flow Cytometer (BD), and data were evaluated using the FlowJo software v. 7.6.1.

*In vitro migration assay:* Hair matrix cell suspensions were prepared and seeded with Culture-Insert (Ibidi 80206, American) into each plate. Then, 70ul of cell suspension was placed into each well. The inserts were incubated at 37 ℃ in 5% CO2, and were removed with sterile tweezers after 24h. The used wells were filled with DMEM and after treatment with 40 µg/well DP-EVs, DF-EVs, KC-EVs or PBS, the wound area was imaged with a reverse phase-contrast microscope (IX61 FL, Olympus, Japan) immediately, after 12h, 24h and 48h. We then generated quantitative analysis data with Wim-Scratch.

*Immunofluorescence of hair matrix cells:* Hair matrix cells were fixed with 4% paraformaldehyde for 15 minutes, washed three times with PBST, and permeabilized with 0.1% Triton X-100 for 10 minutes at room temperature. After blocking with 5% BSA for 40 minutes, cells were incubated with a 1:500 dilution of Ki67 antibody (Abcam) overnight at 4 °C, and subsequently with a 1:300 dilution of Alexa Fluor 594-conjugated anti-rabbit IgG or Alexa Fluor 488-conjugated anti-rabbit IgG antibody (Abcam) for 2 hours. Coverslips were mounted on glass slides using ProLong® Diamond Antifade Mountant with DAPI (Life Technologies, USA) prior to imaging with a confocal laser scanning microscope.

*Preparation of OSA-EV hydrogel:* EVs were isolated from low-passage (P1-P3) hDPC culture by ultracentrifugation, and OSA (4.95% oxidation) was prepared as described above. A mixture of 100 µl OSA (3 g/ml) and 100 µl EV solution (500 µg) was prepared to yield the following final concentrations: 1.5 g/ml OSA and 2.5 mg/ml EVs. An Eppendorf TransferMan NK2 was used for nanosphere construction. The paste was gently extruded dropwise through a microinjection Femtotip (40 µm inner diameter) into a cell culture dish covered with 0.1 M CaCl_2_ crosslinking solution, and cake-like particles formed instantaneously. After hardening for 30 minutes, the OSA-EV solution could crosslink Ca^2+^ into hydrogel. Finally, the solution was washed three times with PBS to remove any residual CaCl_2_.

*Encapsulation and drug loading efficiency of OSA hydrogel:* After construction of OSA-EVs with a specific number of EVs (W_0_), samples were dried at 70 °C and their weight (W_n_) was determined with an electronic microbalance (Mettler-Toledo). Dried microgels were mixed in PBS and incubated with a magnetic stirrer for 2 hours, followed by ultrasonic irradiation at 521 kHz (Tektronix, UK) for 90 minutes to break up the nanofilm. The sample was then centrifuged at 2000 × *g* for 20 minutes, supernatant was removed and replaced with fresh PBS, and the sample was centrifuged again. The sediment, which consisted of EVs encapsulated in OSA hydrogel (W_e_), was collected and analyzed using a BCA total protein assay. The encapsulation and drug loading efficiency were calculated using the formulas below:

Encapsulation efficiency (%) = W_e_/W_0_ × 100

Drug loading efficiency (%) = W_e_/W_n_ × 100

where W_e_ represents the weight of encapsulated EVs, W_0_ represents the weight of EVs used for OSA-EV construction, and W_n_ represents the weight of dried OSA-EV microgels.

*Degradation of OSA-EV hydrogel:* OSA-EVs (40 µg) were added to a 48-well plate and incubated in 5% CO_2_ at 37 °C. The number of remaining microgels and their volumes were determined each day for 7 days using confocal laser scanning microscopy and SEM.

*Thermal stability of DP-EVs and OSA-EVs:* OSA-EVs (containing 40 µg EVs per well) or EVs (40 µg per well) were added to four wells of a 48-well plate and incubated in 5% CO_2_ at 37 °C. The amount of total protein was measured immediately and after 48, 96, 144 and 192 hours. Total vesicular protein was quantified using a BCA assay, as described above. Before measuring the total protein in OSA-EVs, the OSA hydrogel scaffold was removed using ultrasound, as described previously 66,67. Briefly, OSA-EV hydrogel was transferred to the ultrasound reaction vessel (Tektronix), then ultrasonic irradiation (Tektronix) was carried out at 521 kHz for 90 minutes. The sample was then centrifuged at 200 × *g* for 20 minutes and the pellet was discarded. RIPA protein lysis buffer containing 1 mM PMSF was added, and total protein was quantified using a BCA protein assay kit.

*Dynamic release:* OSA-EV hydrogel in PBS was added to 3.0 µm Transwell chambers in a 24-well plate containing cultured hair matrix cells at 37 °C. The released protein was measured on days 0-9 by collecting the supernatant and measuring the total protein it contained (expressed as W_i_, i = 0, 24, 48,…, 192) using a BCA protein assay. The collected supernatant was replaced with fresh PBS. Powder matrix samples were subjected to ultrasonic irradiation (521 kHz for 90 minutes) and centrifugation (200 × *g* for 20 minutes) prior to quantifying vesicular protein. The matrices were recovered and washed three times with PBS, and the residual protein amount (W_r_) was quantified. The dynamic release of total protein (DRP %) and accumulative release of total protein (ARP %) were calculated using the formulas below:

DRP (%) = W_t_/(W_0_+…+W_192_+W_r_) × 100

ARP (%) = (W_0_+…+W_t_)/(W_0_+…+W_192_+W_r_) × 100

where W_t_ is the weight of vesicular total protein at time t and W_r_ is the weight of residual vesicular protein.

*ELISA:* EVs were placed in a 24-well plate, and OSA-EV hydrogel was placed in 3.0 μm Transwell chambers (ECL TransGen Biotech) to facilitate collection of the supernatant.

Vesicular proteins MMP3 and CD9 were quantified using the ExoELISA-ULTRA kit (System Biosciences Inc., USA) according to the manufacturer's instructions. Before adding to 96-well plates, OSA-EVs were centrifuged at 200 × *g* for 20 minutes and pellets were discarded. RIPA protein lysis buffer containing 1 mM PMSF was added to the supernatants of OSA-EVs and EVs. Standards and samples were incubated for 1 hour at 37 °C, washed three times with 100 µl wash buffer, and incubated with MMP3 or CD9 primary antibody for 10 hours at 4 °C with shaking. Each well was then washed three times with wash buffer, incubated with goat anti-rabbit secondary antibody for 2 hours at room temperature, then washed with PBS for 1 hour with shaking. Standards and samples were incubated with TMB (Solarbio, Beijing, China) for 15 minutes at room temperature and washed three times with wash buffer, followed by addition of 50 µl stop buffer. The absorbance at 450 nm was read immediately using a spectrophotometric plate reader (Varian Company, Australia).

*Uptake of EVs by hair matrix cells:* Prior to the EV uptake assay, the concentration of OSA-EVs was adjusted according to the previously calculated “drug loading efficiency” in order to equalize the effective vesicular concentration of OSA-EVs and DP-EVs. OSA-EV hydrogel containing 40 µg EVs was placed in 0.9 µm Transwell chambers and incubated with hair matrix cells at 37 °C. Cells treated with 40 µg EVs were used as a positive control, and cells treated with OSA-encapsulated DIL without EVs were used as a negative control. The culture medium was replaced every 24 hours, and OSA-EV hydrogels were blown by pipet tip. At 24-hour intervals, hair matrix cells were observed by fluorescence microscopy (IX61 FL; Olympus, Japan). Images were analyzed using Image-Pro Plus. The expression of MMP3 and CD9 by hair matrix cells was quantified by western blotting followed by densitometric analysis.

*Animals:* All experiments were performed using 7-week-old female C57BL/6 mice. Animals were purchased from the Experimental Animal Centre at Southern Medical University (Guangzhou, China). Ethical approval for all experimental procedures was obtained from the Experimental Animal Centre at Southern Medical University.

*In vivo fluorescence imaging:* EVs were labeled with DIL for fluorescence signal tracing as previously described. In the *ex vivo* experiments, human hair follicles were cultured with OSA-EV containing 300 μg EVs, 300 μg DP-EVs, or 0.1% DIL 40 μl without EVs for 9 days. For *in vivo* experiments, depilated mice were randomly injected with OSA-EV hydrogel (125 mg/ml dissolved in PBS, 1 ml per mouse), DP-EVs (4 mg/ml dissolved in PBS, 1 ml per mouse), or DIL without EVs (0.1% DIL, 1 ml per mouse) (as a negative control). DIL-labeled EVs were detected using In-Vivo FX Pro imaging system (Bruker, Madison, WI, USA) every 24 hours. All fluorescence intensities were analyzed using the MISE software (Bruker), and are presented in terms of photon flux (photons sec^-1^,cm^-2^steradian [SR]^-1^).

*Human hair follicle samples:* Occipital scalp skin samples were obtained from discarded tissue from elective cosmetic operations on healthy adults (five women aged 35-45 yr). Ethical approval and an institutional review board exemption were obtained from the Medical Ethical Committee of Southern Medical University. Samples were collected in 50-ml sterile universal tubes containing basic culture medium: William's E medium supplemented with 10 ng/ml hydrocortisone, 10 μg/ml insulin, 10 U/ml penicillin, and 2 mM l-glutamine (Life Technologies, Paisley, UK). Unless specified, all materials were supplied by Sigma-Aldrich (Dorset, UK).

*Isolation and culture of human hair follicles:* Hair follicles in anagen stage were microdissected from each sample under an MZ8 dissecting microscope (Leica Microsystems, Wetzlar, Germany). Hair isolation and culture were performed as described by Philpott et al 50. Samples were cut at the level of the sebaceous gland using a scalpel blade. Each isolated follicle was transferred to an individual well of a 24-well plate (Corning Glassworks, Corning, NY, USAA) containing 300 µl of culture medium. Basic culture medium described above was supplemented with OSA-EVs (containing 3, 30, or 300µg DP-EVs), DP-EVs (3, 30, or 300 µg ), DF-EVs (300 µg), KC-EVs (300 µg) or PBS (as negative control) in each well. At least 12 hair follicles from each individual were cultured under each condition, maintained free-floating in an atmosphere of 5% CO_2_ and 95% air at 37℃ in a humidified incubator. Fresh culture medium was added every 3 days.

*Histology and immunofluorescence:* Female C57BL/6 mice aged 7 weeks were divided into four treatment groups (n = 8 for each group): subcutaneous injection with OSA-EV hydrogel (500 μg DP-EVs per mouse every 4 days), subcutaneous injection with DP-EVs (400 μg per mouse every 4 days), external treatment with minoxidil (200 µl per mouse every 4 days), and treatment with PBS every 4 days as a negative control. Animals were examined and photographed every 6 days, and sacrificed at day 21 for histology and immunofluorescence analysis.

The dorsal skin of sacrificed mice was excised and fixed in 4% paraformaldehyde at 4 °C. Samples were stored for less than 1 week. For histology, dorsal skin samples were embedded in paraffin blocks and 5 µm thick sections were prepared according to longitudinal sections of hair follicles, followed by hematoxylin and eosin (HE) staining. Specimens were dehydrated and mounted, then digital photomicrographs of representative areas were obtained at a fixed magnification of 20×. Skin thickness and hair bulb diameter were determined using Image-Pro Plus software.

Immunofluorescence staining of dorsal skin was performed according to the previously described protocol for hair matrix cell immunofluorescence. The following primary antibodies were used at the dilutions indicated: Ki67, 1:100 (Abcam); β-catenin, 1:80 (Abcam); MMP3, 1:200 (Abcam); Wnt3a, 1:200 (Abcam); BMP2, 1:150 (Abcam). Images were obtained using an IX61 FL fluorescence microscope (Olympus) and analyzed using Image-Pro Plus.

*Statistical assessment of hair follicle growth in culture*: Hair follicles were photographed using a Labovert inverted microscope (Leitz Labovert FS, Wetzlar, Germany), assessed for follicle bulb morphology, and subjected to measurement of length every 24 h for 9 days. Hair follicles showing no growth for 3 days were excluded. For each hair growth parameter, the mean value per subject under each treatment was determined before calculation of the corresponding sample mean. Data from each experimental condition were analyzed for normality using the Kolmogorov-Smirnov test. The effect of different treatments with time on the daily rate of growth and percentage of follicles remaining in anagen was analyzed by two-factor, within-subjects ANOVA using the SPSS statistical software (SPSS, Chicago, IL, USAA). If sample means of different experimental conditions differed significantly (P<0.05), they were compared using Student's paired t-test with Sidak's correction for multiple comparisons.

*Quantitative (q)RT-PCR:* Total RNA from mouse dorsal skin was isolated using Trizol reagent (Life Technologies), and cDNA was generated using the PrimeScript RT-PCR Kit (Takara, Dalian, China), according to the manufacturers' instructions. Quantitative (q)RT-PCR was performed using the SYBR PrimeScript RT-PCR Kit (Takara), Power SYBR Green PCR Master Mix (Life Technologies), and an ABI Prism 7900HT Sequence Detection System (Life Technologies). PCR cycling conditions were as follows: denaturation for 10 minutes at 95°C, followed by 40 cycles of denaturation (95°C for 15 seconds), annealing (60°C for 20 seconds), and extension (72°C for 10 seconds). GAPDH expression (4352932E) was used to normalize data using the ΔCt method. Fold changes in relative gene expression were identified using the 2^-ΔΔCt^ method. Primer sequences used in this study are shown in Table S1.

*Statistical analysis:* Statistical analysis was performed using SPSS 14.0 software (SPSS, Inc., Chicago, IL, USA). Experimental data are expressed as the mean ± standard deviation. Data from each experimental condition were analyzed for normality using the Kolmogorov-Smirnov test Comparisons between groups were performed using the Wilcoxon test and *t*-test. *P* < 0.05 was considered statistically significant. Each experiment was repeated at least three times.

## Supplementary Material

Supplementary figures and table.

## Figures and Tables

**Table 1:** Characterization of oxidized sodium alginates.

Sample	Guluronate unit/ periodate (mol/mol)	Degree of oxidation (%)	M_η_ (×10^5^)
SA	**-**	**-**	4.40
5% OSA	100:5	4.95	3.28
7.5% OSA	100:7.5	7.47	2.64
10% OSA	100:10	9.90	2.31
20% OSA	100:20	18.48	1.98
30% OSA	100:30	28.84	1.27

OSA: oxidized sodium alginate

**Table 2:** Encapsulation and drug loading efficiency of 5% hydrogel.

Vesicular concentration	Encapsulation efficiency (%)	Drug loading efficiency (%)
2.5mg/ml	78.5%	4.3%

## Abbreviations

DO: degree of oxidation
DP: dermal papilla
DPCs: dermal papilla cells
DP-EVs: dermal papilla-derived EVs
EVs: extracellular vesicles
FTIR: fourier transform infrared
hDPCs: human dermal papilla cells
miRNAs: microRNAs
MMP3: matrix metallopeptidase 3
MSCs: mesenchymal stem cells
NTA: nanoparticle tracking analysis
OSA: oxidized sodium alginate
OSA-EVs: oxidized sodium alginate-encapsulated EVs
P3: passage 3
SR: swelling ratio
TGF-β: transforming growth factor-beta
WNT: wingless-type MMTV integration site family
3D: three-dimensional
