# Supplementary material for: Sustained release of dermal papilla-derived extracellular vesicles from injectable microgel promotes hair growth
Source: Theranostics. 2020 Jan 1;10(3):1454–78. doi: 10.7150/thno.39566 (PMC6956798; doi:10.7150/thno.39566)
Supplement: Supplementary file 1 — Supplementary figures and table. [file thnov10p1454s1.pdf]

Supporting Figures

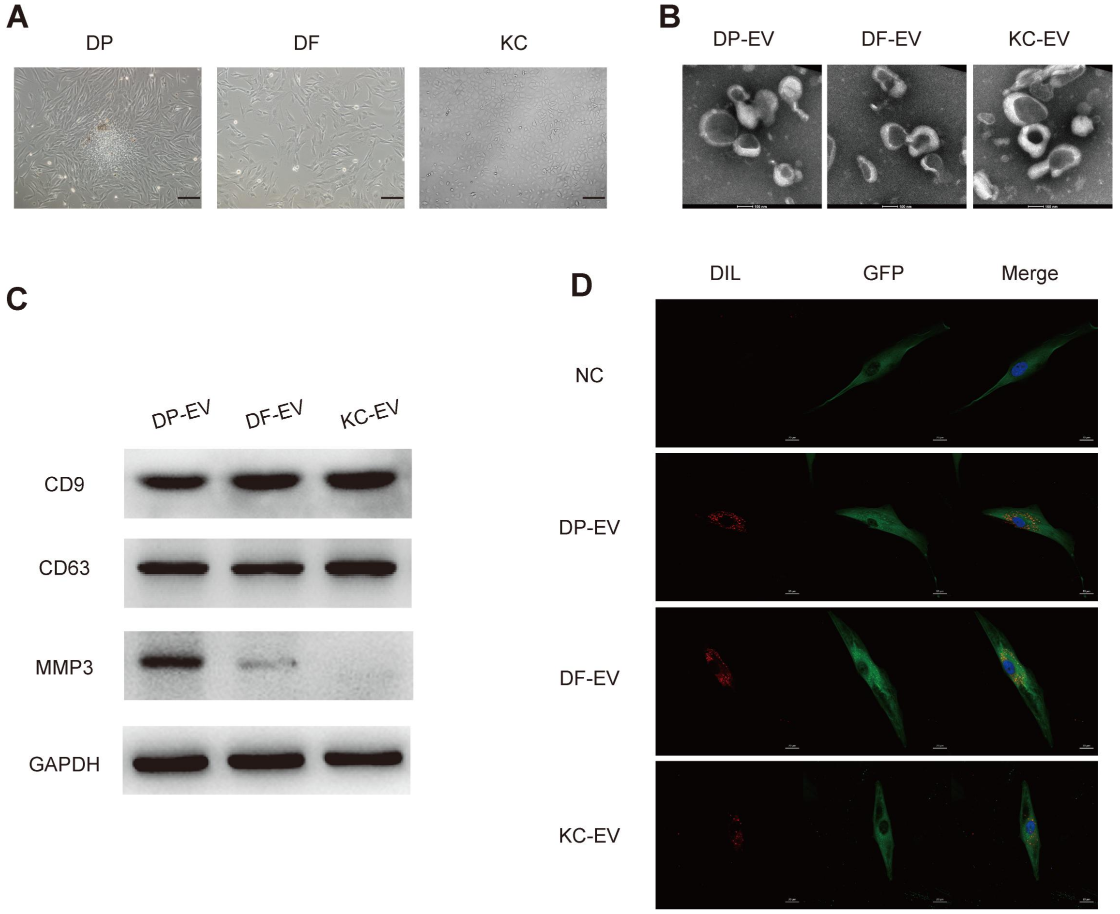

Figure S1. Isolation and identification of EVs from different cells in the skin. (A) micrographs of DPCs, DFs and KCs in P3. Scale bars: 50 μm. (B) Observation of EVs derived from DPCs (DP-EVs), DFs (DF-EVs) and KCs (KC-EVs) respectively by transmission electron microscopy. Scale bar, 100nm. (C) Western blot of CD9, CD63, and MMP3 in DP-EVs, DF-EV, and KC-Exos respectively. (D) DIL-labeled EVs derived from DPCs (DP-EVs), DFs (DF-EVs) and KCs (KC-EVs) were taken up by hair matrix cells, PBS was used as negative control. Scale bar, 20 μm. NC: negative control.

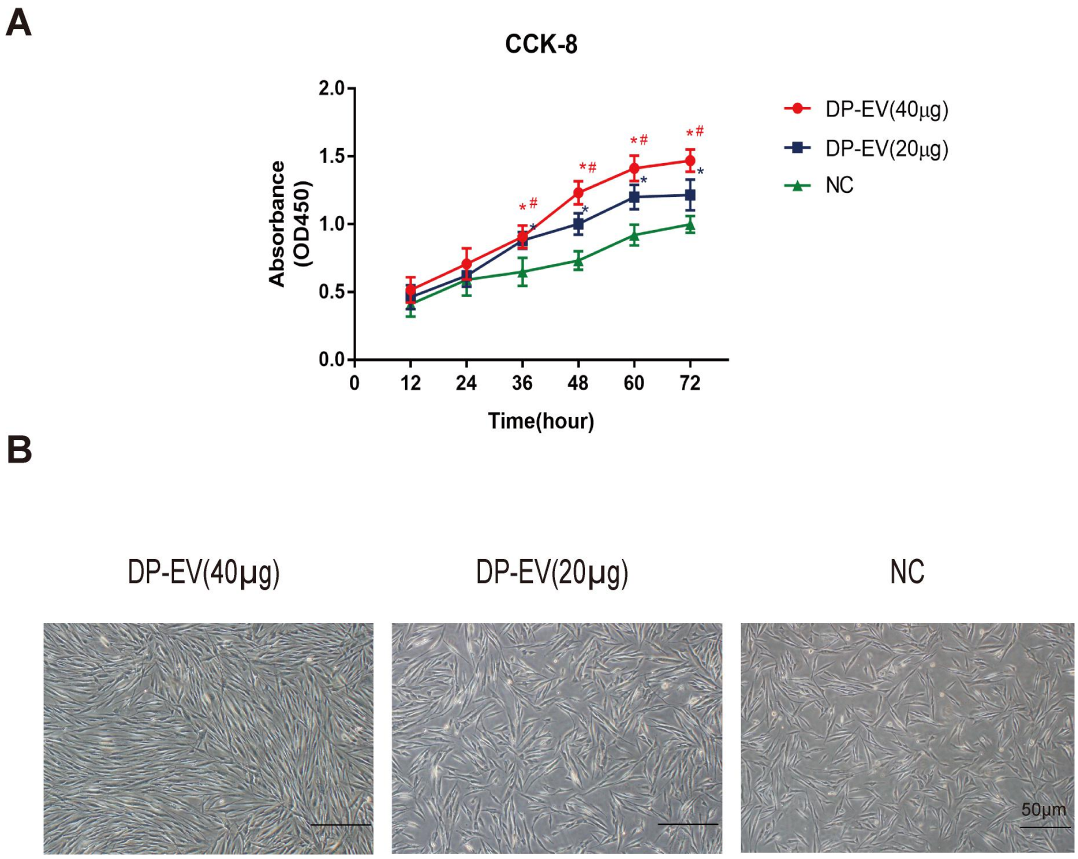

Figure S2. DPC-EVs stimulate proliferation of hair matrix cells.(A) Hair matrix cells were cultured with 20 μg DP-EVs, 40 μg DP-EVs, or PBS (as a negative control), and proliferation was evaluated by CCK-8 assay every 12 hours. (B) Micrographs of hair matrix cells after 48 hours of treatment with 20 μg DP-EVs, 40 μg DP-EVs, or PBS.

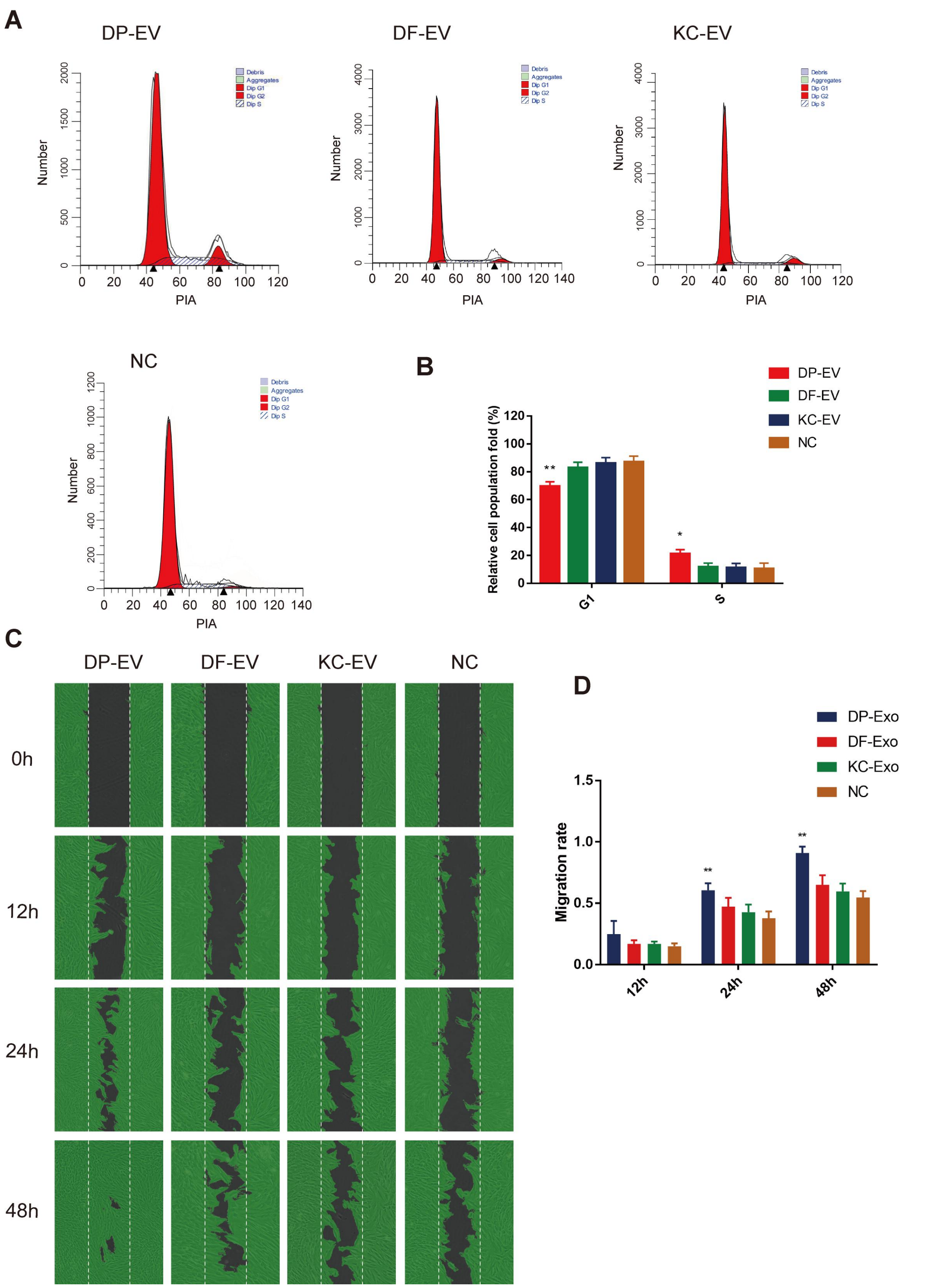

Figure S3. DP-EV stimulated hair matrix cells proliferation and promoted cell migration. (A) Cell cycle analysis of hair matrix cell proliferation after treating with DP-EVs, DF-EVs and KC-EVs for 48h respectively. (B) Quantification of hair matrix cells in G1 and S phases in the presence or absence of EVs. (C) Representative images of hair matrix cells in the wounded area during the migration assay, after incubation for 0, 12, 24 h and 48h with 40 ug DP-EVs, DF-EVs, KC-EVs or PBS respectively. (D) Quantitative analysis of the decrease in wound area in Figure

S2C. *. indicates a statistically significant difference ($p < 0.05$), compared to NC; ** indicates a statistically significant difference ($p < 0.01$), compared to NC; all values are expressed as means ± S.D. (n = 3 individual experiments).

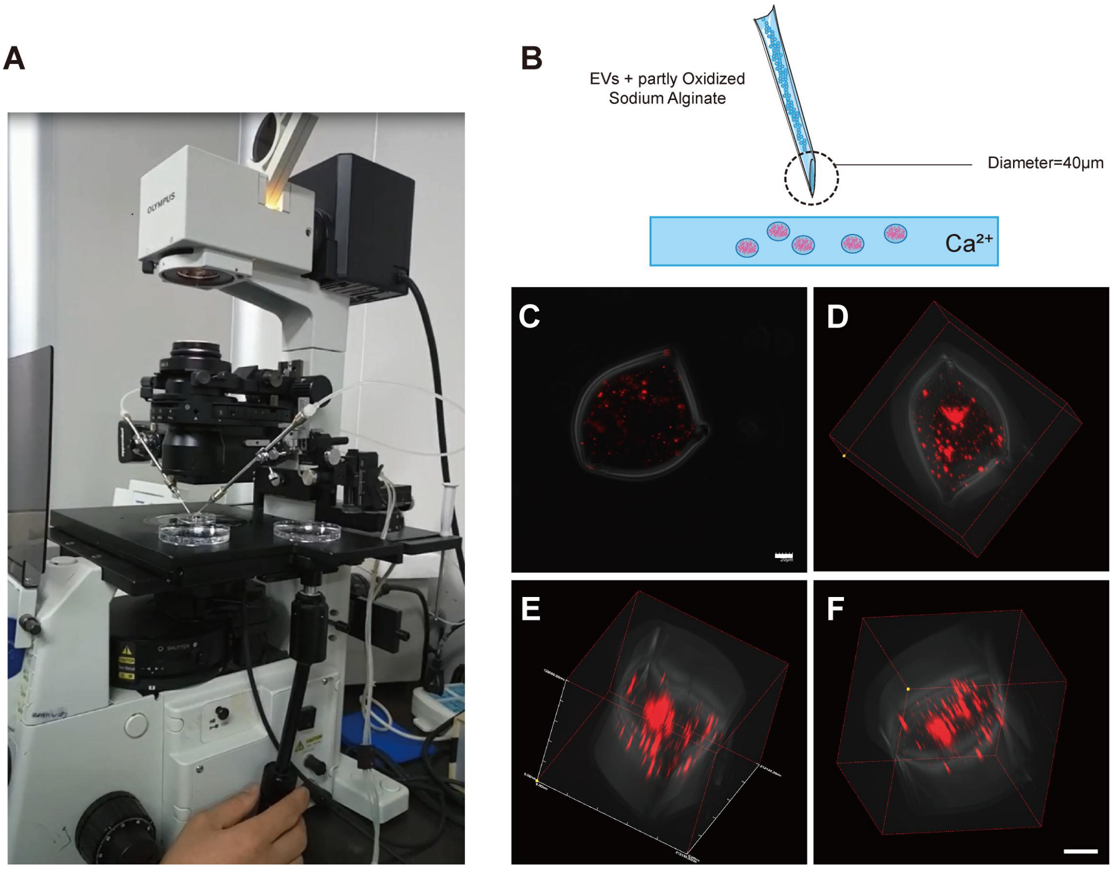

Figure S4. Preparation of injectable OSA-EV hydrogel nanospheres. (A) Eppendorf TransferMan NK2(Hamburg, Germany) for construction of OSA-EV nanospheres.(B) Schematic pictures of forming OSA-EV hydrogel nanospheres: OSA solution mixed with DP-EVs were dropped into calcium chloride solution (0.1 M) with a microinjection Femtotip (inner diameter, 40 μm) and incubated for 15 minutes to initiate gelation.(C) Confocal micrographs of OSA-EV nanospheres. DP-EVs were labeled with DIL (red) and coated with OSA hydrogel. Scale bars: 20 μm. (D–F) 3D reconstruction of OSA-EV nanosphere, projected from various angles. Scale bars: 10 μm.

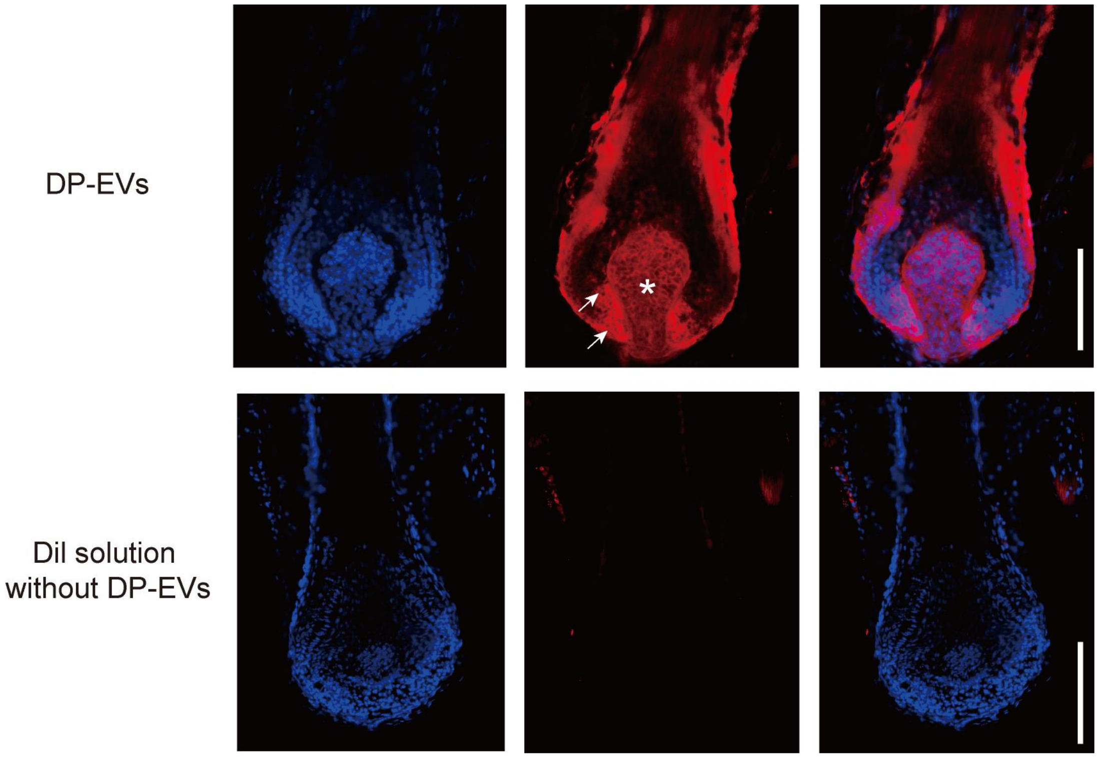

Figure S5. DP-EVs settle in cultured hair follicles. Human scalp hair follicles were cultured with DIL-labeled DP-EVs or DIL solution without EVs. After 24 hours of culture, dermal papilla (star) and hair matrix (arrow) in follicles cultured with DP-EVs contained red staining. However, follicles cultured with DIL solution without EVs contained no staining. Scale bar: 200 μm.

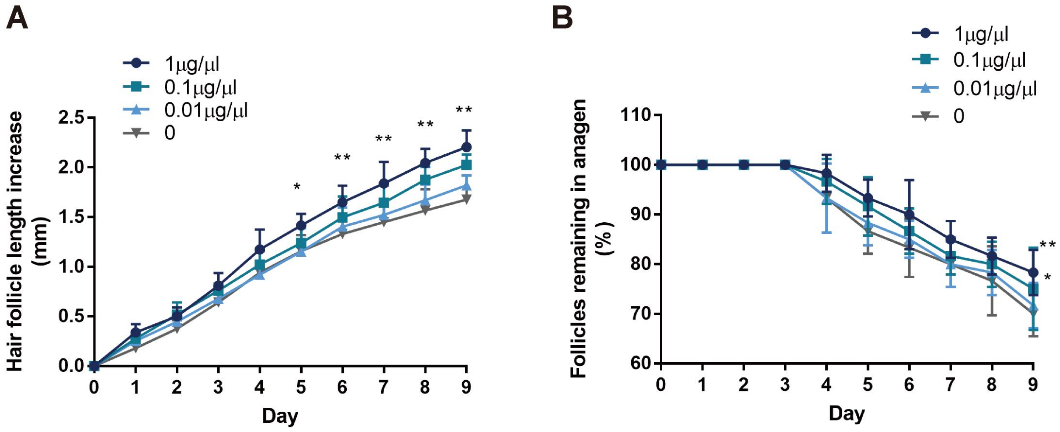

Figure S6. DP-EVs stimulate scalp hair follicle growth in organ culture in a concentration-dependent manner. (A) Hair follicles were treated with 1 μg/μl, 0.1 μg/μl, or 0.01 μg/μl DP-EVs. At a concentration of 1 μg/μl, DP-EVs significantly increased hair follicle growth ($P<0.01$) (B) Percentage of follicles remaining in anagen upon treatment with the indicated concentrations of DP-EVs. Data are expressed as means ± s.d. Five individuals were used each experiment, and 12 follicles/person were examined for each condition. * $p < 0.05$ relative to the control; ** $p < 0.01$. Statistical significance was determined by ANOVA with Bonferroni correction.

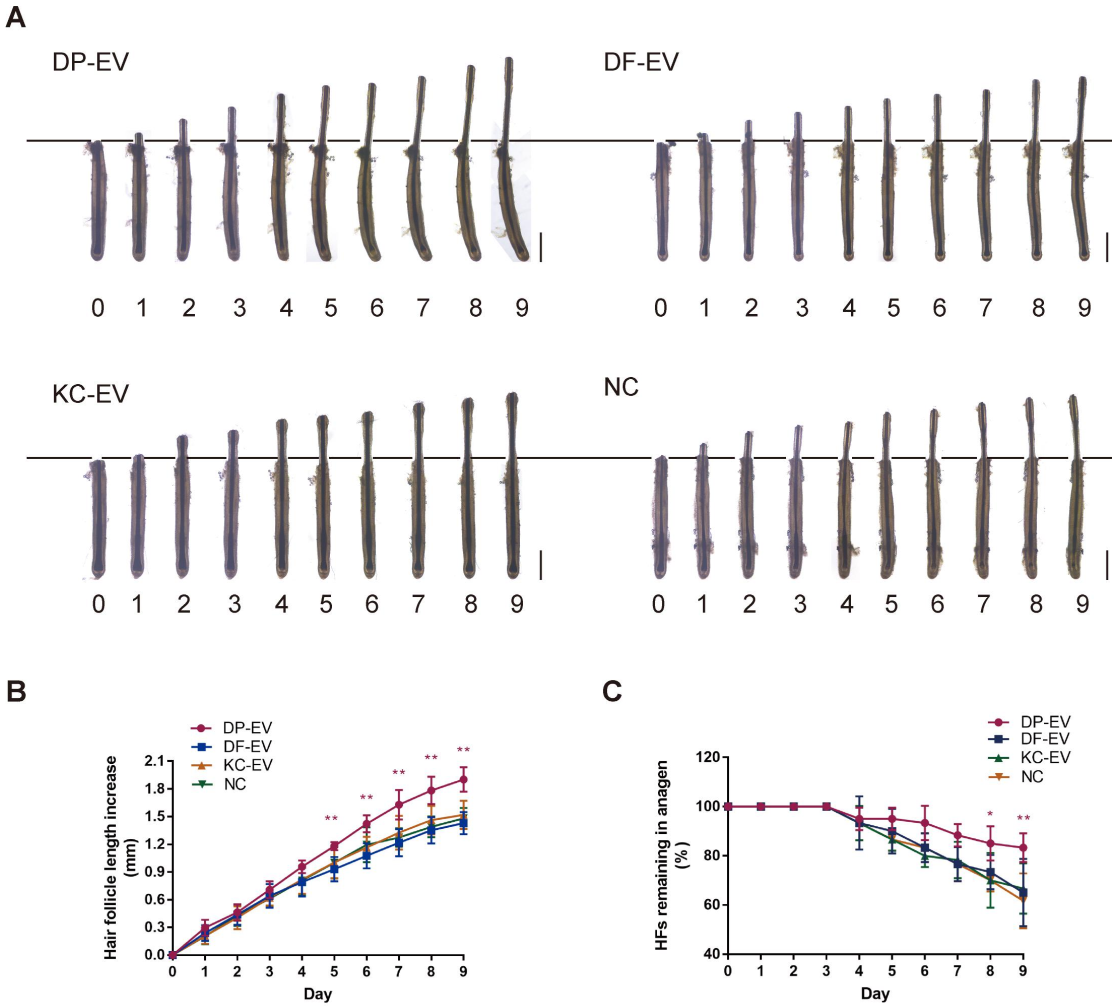

Figure S7. DP-EVs promoted human hair growth ex vivo. (A) Individual HFs were dissected from adult human scalp tissue and placed in culture in the presence of 1 μg/μl DP-EVs, DF-EVs, KC-EVs, or PBS. Photomicrographs were taken every 24 h for 9 d. (B-C) Hair follicles in each group were assessed and measured daily for increase in hair length (B), and the percentage of hair follicles remaining in anagen (C). * indicates a statistically significant difference ($p < 0.05$), compared to NC; ** indicates a statistically significant difference ($p < 0.01$), compared to NC; all values are expressed as means ± S.D. (n = 60 individual experiments).

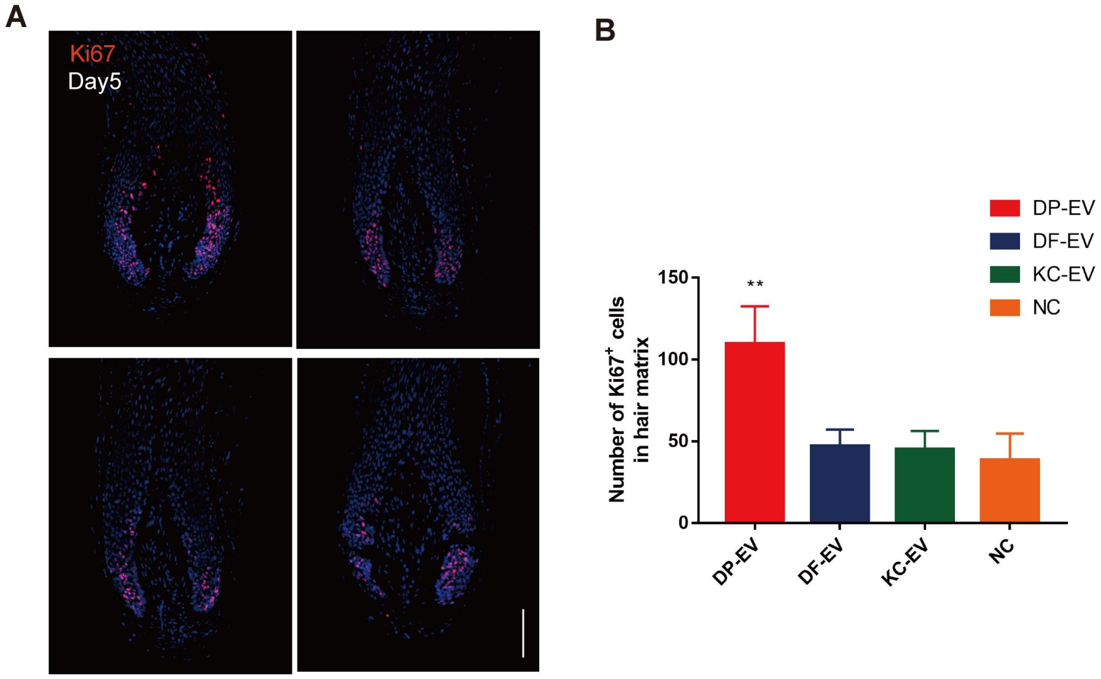

Figure S8. DP-EVs promoted hair matrix cells proliferation in cultured human HFs. (A) Immunofluorescence analysis of Ki67^{+} cells (red) in hair matrix at day 5 in DP-EV-treated, DF-EV-treated, KC-EV-treated and PBS-treated group. Scale bars: 50 μm. (B) Quantitative analysis of the number of Ki67^{+} cells in hair matrix in each group. Data are expressed as mean ± s.d. Five individuals were used per experiment and at least 12 follicles/person were examined for each condition. ** indicates a statistically significant difference ($p < 0.01$), compared to NC; all values are expressed as means ± S.D. ($n = 60$ individual experiments).

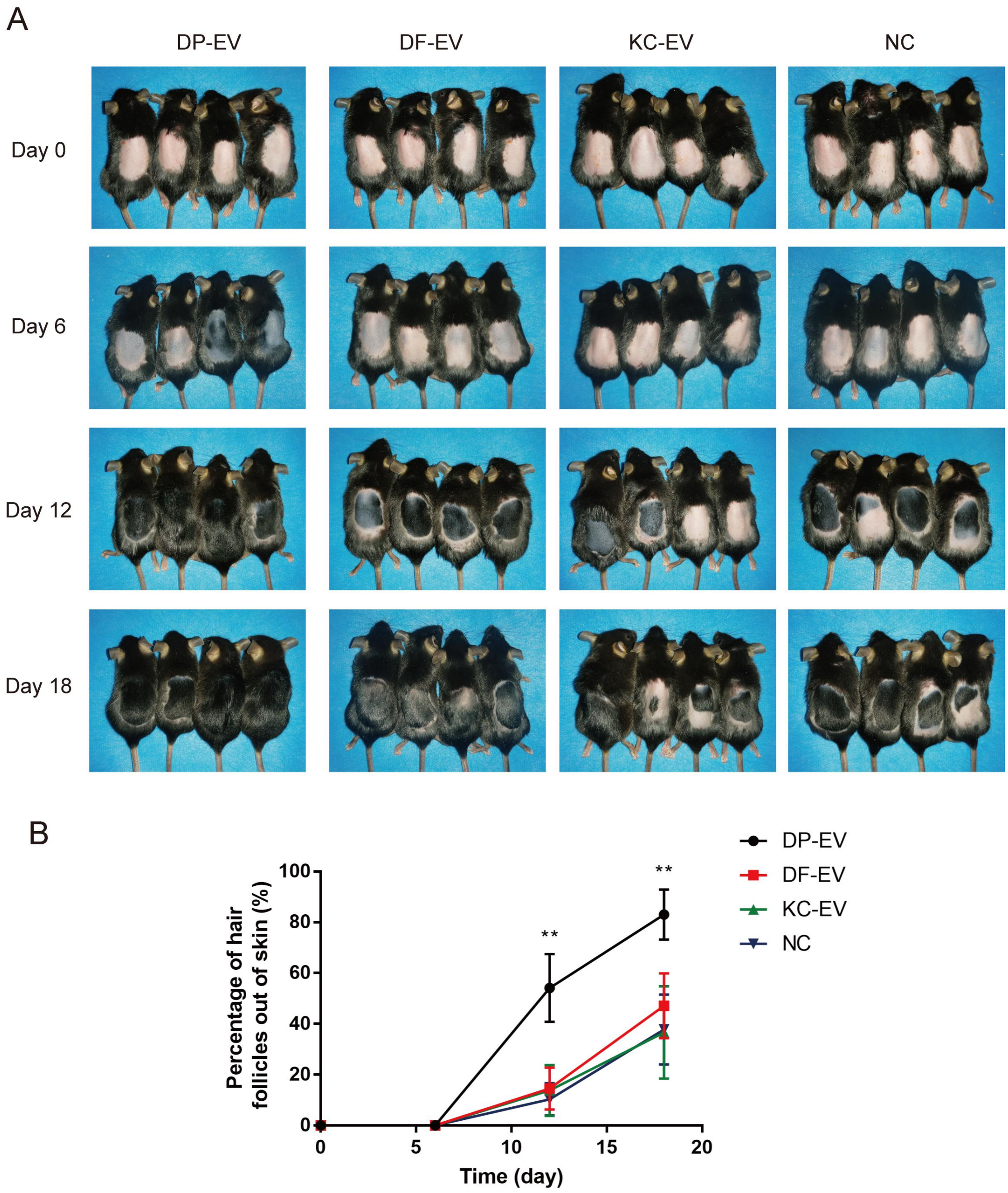

Figure S9. DP-EVs accelerated the telogen-to-anagen transition in C57BL/6 mice. (A) After hair depilation, mice dorsal skin was treated every 4 days with DP-EVs, DF-EVs, KC-EVs , or PBS. Photomicrographs were taken every 6 days for 18 days. (B) Areas with hair regrowth out of skin were quantified using Image-Pro Plus software.** indicates a statistically significant difference ($p < 0.01$), compared to NC; all values are expressed as means ± S.D. ($n = 32$, every individual experiments).

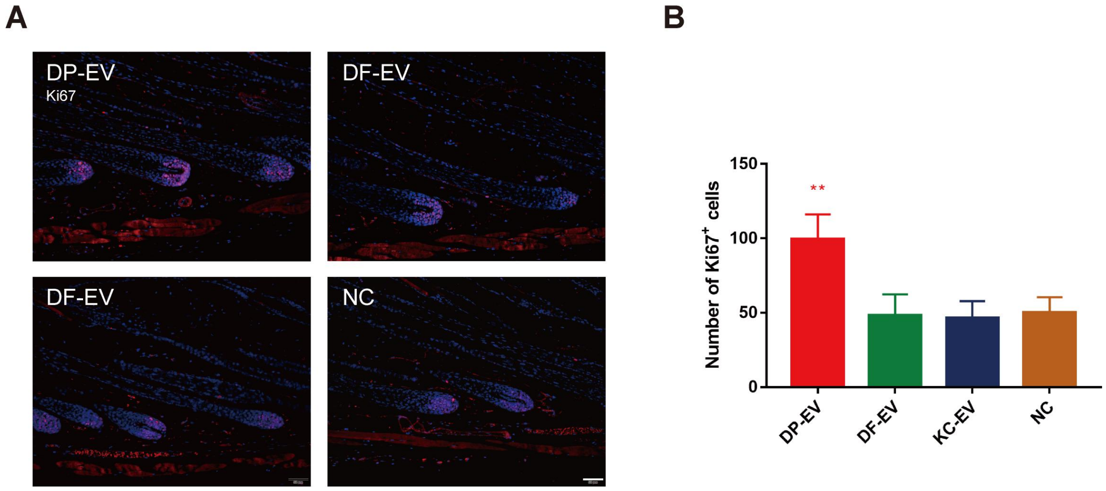

Figure S10. DP-EVs stimulated hair matrix cell proliferation in C57BL/6 mice. (A) Immunofluorescence staining of Ki67$^+$ cells in dorsal skin on day 21. Scale bars: 50 µm. (B) Quantitative analysis of the number of Ki67$^+$ cells in each group.** indicates a statistically significant difference ($p < 0.01$), compared to NC; all values are expressed as means ± S.D. (n = 32, every individual experiments).

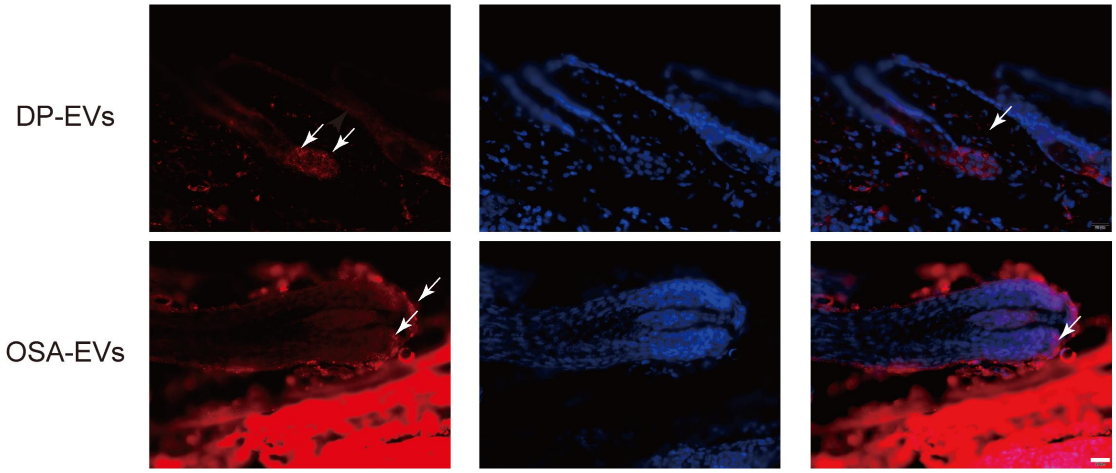

Figure S11. DP-EVs settle in hair follicles of C57BL/6 mice. Following hair depilation, C57BL/6 mice were subcutaneously injected with DIL-labeled DP-EVs or OSA-EVs. Forty-eight hours after treatment, dorsal skin in paraffin sections was examined by fluorescence microscopy. Scale bar: 20 µm.

Supporting Table

Table S1. Primer pairs for qRT-PCR

β-catenin	β-catenin-F	CGTGAAATTCTTGGCTATTACAAC
	β-catenin-R	AGACAGACAGCACCTTCAGCA
Wnt3a	Wnt3a-F	GCAGAGGGCATCAAAATC
	Wnt3a-R	CTGTTGTTGATGGTGGTG
MMP3	MMP3-F	TCGTTGCTGCTCATGAAATTG
	MMP3-R	AGCTTCAGTGTTGGCTGAGTGA
BMP2	BMP2-F	CTTCAGTGATGTGGGGTGGA
	BMP2-R	TGGTTAGTGGAGTTCAGGTGGT
Actin	Actin-F	CTGAGAGGGAAATCGTGCGT
	Actin-R	CCACAGGATTCCATACCCAAGA
